# Patients' perceived needs for allied health, and complementary and alternative medicines for low back pain: A systematic scoping review

**DOI:** 10.1111/hex.12676

**Published:** 2018-07-07

**Authors:** Louisa Chou, Tom A. Ranger, Waruna Peiris, Flavia M. Cicuttini, Donna M. Urquhart, Andrew M. Briggs, Anita E. Wluka

**Affiliations:** ^1^ Department of Epidemiology and Preventative Medicine School of Public Health and Preventative Medicine Monash University Melbourne Vic Australia; ^2^ School of Physiotherapy and Exercise Science Curtin University Perth WA Australia; ^3^ Move: Muscle, Bone & Joint Health Melbourne Vic Australia

**Keywords:** allied health, complementary therapies, low back pain, needs assessment, systematic review

## Abstract

**Objectives:**

Allied health and complementary and alternative medicines (CAM) are therapeutic therapies commonly accessed by consumers to manage low back pain (LBP). We aimed to identify the literature regarding patients' perceived needs for physiotherapy, chiropractic therapy and CAM for the management of LBP.

**Methods:**

A systematic scoping review of MEDLINE, EMBASE, CINAHL and PsycINFO (1990‐2016) was conducted to identify studies examining patients' perceived needs for allied health and CAM for LBP. Data regarding study design and methodology were extracted. Areas of patients' perceived need for allied health and CAM were aggregated.

**Results:**

Forty‐four studies from 2202 were included: 25 qualitative, 18 quantitative and 1 mixed‐methods study. Three areas of need emerged: (i) physiotherapy was viewed as important, particularly when individually tailored. However, patients had concerns about adherence, adverse outcomes and correct exercise technique. (ii) Chiropractic therapy was perceived to be effective and needed by some patients, but others were concerned about adverse outcomes. (iii) An inconsistent need for CAM was identified with some patients perceiving a need, while others questioning the legitimacy and short‐term duration of these therapies.

**Conclusions:**

Our findings regarding patients' perceived needs for allied health and CAM for LBP may assist in informing development of more patient‐centred guidelines and service models for LBP. Understanding patients' concerns regarding active‐based physiotherapy, which is recommended in most guidelines, and issues surrounding chiropractic and CAM, which are generally not, may help inform management that better aligns patient's perceived needs with effective treatments, to improve outcomes for both patients and the health‐care system.

## 1 INTRODUCTION

Low back pain (LBP) is a major public health problem and has been identified as the leading cause of disability worldwide.^1^ Approximately 80% of adults experience at least one episode of LBP during their lifetime.^2^ One in five adults and adolescents experience persistent LBP symptoms.^2^ Persistent LBP is associated with significant individual functional impairment, high utilization of health care, work absenteeism and long‐term incapacity.^3^ The economic burden of LBP is substantial and was estimated to exceed $US100 billion per year according to a review performed in 2006.^4^

To address this problem, evidence‐based guidelines have been developed to optimize treatment outcomes for people with LBP. Most clinical practice guidelines for chronic LBP recommend patient education, supervised exercises, multidisciplinary treatment and cognitive behavioural therapy, as well as short‐term use of pharmacological therapies such as paracetamol, non‐steroidal anti‐inflammatory drugs and weak opioids.^5^, ^6^, ^7^ Currently, the guidelines do not recommend the use of chiropractic therapies as unimodal or long‐term interventions or complementary and alternative medicines (CAM), based on inconclusive and conflicting evidence regarding efficacy and potential risk of harm.^5^, ^6^, ^7^, ^8^ CAM therapies are heterogeneous and include a range of diagnostic and therapeutic modalities that lie outside of conventional health care in Western societies, but may be more mainstream in other settings.^9^ Despite the lack of evidence, population‐based studies have found that approximately a third of patients with LBP visit CAM practitioners and 45% seek care from chiropractors.^10^, ^11^ The high prevalence of CAM use among patients with LBP mirrors that of other chronic illnesses such as arthritis, cardiovascular disease, asthma and diabetes.^12^, ^13^ In this situation, it may be due to the high level of patient dissatisfaction with LBP management from medical practitioners,^14^, ^15^ a strong desire for pain relief that is not achieved through other treatment options in a timeframe acceptable to patients,^16^ information from peers or other providers or a preference for passive therapies, rather than an active approach to LBP care. Furthermore, alignment of clinical practice with guidelines is suboptimal with <50% of patients with LBP being referred for active rehabilitation strategies, despite these being recommended in all guidelines, including the most recent NICE guidelines.^17^, ^18^

Currently, the utilization of health services for LBP does not adequately mirror recommendations in clinical practice guidelines or models of care. The uptake of clinical practice guidelines depends on a complex interplay of factors related to patients (micro level), health‐care providers/organizations (meso‐level) and health systems (macro level). While the suboptimal utilization of LBP clinical guidelines may be related to resource restraints at the level of health systems, or physician factors such as a lack of knowledge of recommended guidelines, the patient plays a pivotal role in use of guidelines and evidence‐based medicine generally^19^, ^20^, ^21^, ^22^ The patient ultimately decides which services they will use. LBP guidelines have largely been developed by health‐care professionals, who determine the primary outcomes, whereas the need for and success of health‐care interventions may be differently perceived by the patient.^23^ Therefore, identifying and understanding the patient perspective and their perceived needs of health services for LBP may provide insight into suboptimal patient uptake of, or adherence to, best‐practice care for LBP. Identifying where patients' needs align with best practice and where and why they deviate may inform more effective patient‐centred service delivery models. Within the context of physical therapies for LBP management, physiotherapy, chiropractic and some CAM modalities are the most frequently accessed treatment modalities.^24^ Therefore, we aimed to conduct a systematic scoping review to provide a broad overview of the existing literature regarding patient perceived needs of physiotherapy, chiropractic therapy and CAM for LBP. Given the breadth of this topic, this review examines the perceived needs of allied health and CAM and does not cover medical services.

## 2 METHODS

We performed a systematic review to identify what is known about patients' perceived needs of physiotherapy, chiropractic therapy and CAM for LBP within a larger project examining the patients' perceived needs relating to musculoskeletal health.^25^ A systematic scoping review was performed to enable a comprehensive exploration of the patients' perspective, map the existing literature and identify gaps in the evidence.^26^, ^27^

### 2.1 Search strategy and study selection

An electronic literature search was performed of relevant databases (MEDLINE, EMBASE, CINAHL and PsycINFO) between January 1990 and June 2016. The time period (1990‐2016) was chosen to include studies relevant to the current patient perspective. A comprehensive search strategy was developed iteratively by a multidisciplinary team involving an academic librarian, patient input and clinician researchers (rheumatologists and physiotherapists). The search strategy combined both MeSH terms and text words. Based on the outcomes of the search, we grouped the data broadly according to discipline, rather than specific intervention as most of the data in the included studies referred to disciplines, rather than interventions. To ensure objectivity in this framework, we developed operational definitions of each discipline to accommodate studies where specific interventions were reported. We have used the term “physiotherapy” to capture therapeutic exercise, general exercise or physical activity guided or prescribed by a physiotherapist, manual therapies, education, or other physical therapies or aids commonly applied or used by a physiotherapist. The term “chiropractic therapy” refers to spinal and joint manipulation delivered by chiropractors. The term “CAM” incorporates a variety of healing resources including acupuncture, homoeopathy, osteopathy, massage therapy, reflexology, heat therapy, naturopathy, traditional Chinese Medicine and Reiki. There is potential for overlap between the treatment modalities offered between the disciplines of physiotherapy, chiropractic therapy and CAM. The detailed search strategy is provided in the Appendix S1.

Between three authors, LC (consultant Rheumatologist), TR (physiotherapist) and WP (PhD Candidate), the results of the search strategies were reviewed independently and in duplicate for relevance. The initial screening was set to be open‐ended, and all study designs were included to retain as many relevant studies as possible. Studies were included if they met the inclusion criteria: (i) patients older than 18 years, (ii) studies had to report on the patients' perspective of needs of physiotherapy, chiropractic and CAM for LBP and (iii) patients with LBP with or without leg pain, excluding LBP from fractures, malignancy, infection and inflammatory spinal disorders. Patients' perceived needs referred to patients' capacity to benefit from services, including their expectations of, satisfaction with and preferences for various services.^28^ Only human studies in the English language and full‐text articles were included. Those that appeared to meet inclusion criteria were retrieved, and the full text was assessed for relevance. A manual search of the reference lists of the obtained studies and review articles was conducted to identify further studies for inclusion in the review.

### 2.2 Methodological quality assessment

To assess the methodological quality of the included studies, the first author reviewed all of the included studies (LC) while the second review was performed by one of two authors (TR and WP) who independently assessed all the studies. For qualitative studies, the Critical Appraisal Skills Programme (CASP) tool was used.^29^ Hoy et al's^30^ risk of bias tool was utilized to assess the external and internal validity of quantitative studies: low risk of bias of quantitative studies was defined as scoring 8 or more “yes” answers, moderate risk of bias was defined as 6 to 7 “yes” answers, and high risk of bias was defined as 5 or fewer “yes” answers. The reviewers discussed and resolved disagreements through consensus. Any disagreements in scoring were reviewed by the senior author (AW).

### 2.3 Data extraction and analysis

One investigator (LC) extracted the data from relevant studies using a standardized data extraction form developed for this scoping review. The following data were systematically extracted: (i) author and year of publication, (ii) study population (patient age and gender, population source, population size and duration of LBP), (iii) description of the study methods and (iv) primary study aim. Included studies were examined using the principles of meta‐ethnography to synthesize qualitative data.^31^ In the first stage, one author (LC) initially developed a framework of concepts and underlying themes, based on primary data in the studies and any pertinent points raised by the authors in the discussion. These key themes were then reciprocally translated across the included studies. In the second stage, two senior authors (FC and AW) with over 15 years of clinical rheumatology consultant‐level experience and a senior physiotherapist (AMB) independently reviewed the framework of concepts and themes to ensure clinical meaningfulness.

## 3 RESULTS

### 3.1 Overview of articles

**Figure 1:**
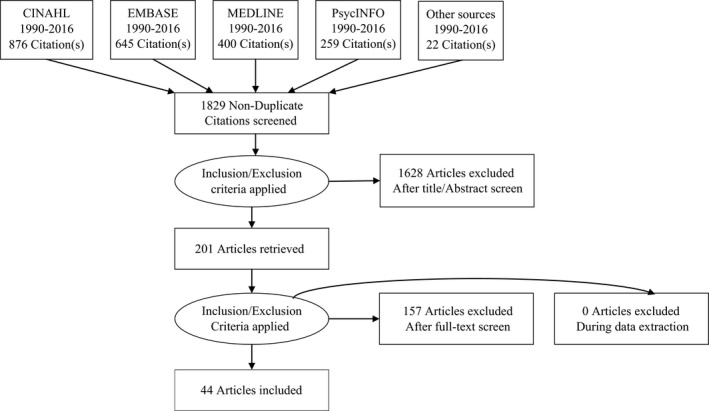
PRISMA diagram of study identification

The search returned 2202 articles, of which 44 studies explored LBP patients' perceived needs of physiotherapy, chiropractic therapy and CAM, based on the discipline definitions we developed. A PRISMA flow diagram detailing the study selection is shown (Figure 1). The descriptive characteristics of the included studies are shown (Table 1). The majority of studies were conducted in the United Kingdom,^14^, ^15^, ^32^, ^33^, ^34^, ^35^, ^36^, ^37^, ^38^, ^39^, ^40^, ^41^, ^42^, ^43^ with the remainder from North America,^11^, ^44^, ^45^, ^46^, ^47^, ^48^, ^49^, ^50^, ^51^, ^52^, ^53^, ^54^, ^55^ Europe,^56^, ^57^, ^58^, ^59^, ^60^, ^61^, ^62^, ^63^, ^64^ Australasia,^65^, ^66^, ^67^, ^68^, ^69^, ^70^ Middle East^71^ and Asia.^72^

**Table 1:** Studies identified in the systematic review of patients' perceived health service needs related to LBP

Author, year & country	Diagnosis of back pain	Participants	Source of participants	Age and gender	Primary study aim	Study design
Allegretti (2010) 46 USA	Chronic LBP (>6 mo of daily or near daily pain)	23	Purposeful sample from Family Care Centre, Memorial Hospital	Average age 45 (28‐72) 52% female	“To explore discrepancies between patients with chronic LBP and physicians using paired interviews of shared experiences aiming to improve doctor‐patient communication and clinical outcomes”^46^	Qualitative: In‐depth interviews
Amonkar (2011) 14 UK	Duration of LBP not specified 46.2% of men had a history of LBP and 49.4% of women had a history of LBP	533	50 consecutive patients were recruited from 12 GP practices.	Age distribution not specified 63% Female	“To investigate whether doctors and patients have different perceptions and expectations with respect to the management of simple chronic back pain”^14^	Quantitative: Questionnaires
Astin (1998) 47 USA	Duration of LBP not specified	1035	Random sample from a representative national sample of US persons.	Age 18‐24 yo 7.9%, 25‐34 yo 21.5%, 35‐49 yo 34.8%, 50‐64 yo 18%, >64 yo 7.9% 51% female	“To investigate possible predictors of alternative health care use”^47^	Quantitative: Mail survey
Borkan (1995) 71 Israel	At least 1 episode of LBP (patients not included on basis of intensity/duration of pain) Duration of LBP not specified	66	10 focus groups, 3 geographic locations from family medicine practices. Participants were identified by community nurses, physicians or through chart review (purposive recruitment)	Average age 39.5 (range 18‐67) 35% female	“To increase the understanding of LBP through access to patients' perceptions, beliefs, illness behaviours and lived experiences”^71^	Qualitative: Focus groups, individual interviews and participant observation
Campbell (2007) 33 UK	LBP >1 y	16	Patients who had completed a Pain Management Program and requested further secondary care referrals for continuing pain.	Age range 34‐78 Gender of patients not specified	“To examine expectations for pain treatment and outcome and to determine whether they are influential in maintaining health service consumption”^33^	Qualitative: Group discussions
Carey (1995) 49 USA	LBP <10 wk duration	1555	208 practitioners in North Carolina were randomly selected from 6 strata (urban primary care physicians, rural primary care physicians, urban chiropractors, rural chiropractors, orthopaedic surgeons and primary care providers) and asked to enrol consecutive patients with acute LBP.	Urban primary care physician: mean age 41, 66% female Rural primary care physician: mean age 43, 57% female Urban chiropractor: mean age 40, 50% female Rural chiropractor: mean age 44, 45% female Orthopaedics: mean age 40, 48% female Health maintenance organization: mean age 38, 58% female	“To determine whether the outcomes of any charges for care differ among primary care practitioners, chiropractors and orthopaedic surgeons”^49^	Quantitative: Interviews and telephone surveys
Carey (1996) 48 USA	Severe LBP—defined as back pain that leads to the respondent's being unable to perform his or her usual daily activities LBP (functionally limiting pain <3 mo)	485	Participants with LBP were recruited by stratified sampling of telephone numbers.	Patients seeing doctors: 19% of patients were >60 yo and 64% female Patients seeing chiropractors: 5% were >60 yo and 27% female	“To examine correlates of care‐seeking in people with LBP”^48^	Quantitative: Telephone interviews
Carey (1999) 50 USA	Recurrence of back pain	754	Practitioners were randomly selected from medical and chiropractic state licensure files from 6 strata (see above study in 1995).^49^ Practitioners invited sequential patients with acute LBP to participate.	Mean age 41.7 51% female	“To explore the relationship between type of initial care as well as the likelihood of recurrence and consequent care seeking behavior”^50^	Quantitative: Telephone interviews
Chen (2015) 72 China	Chronic LBP, duration not specified	86	Participants recruited from the outpatient clinics and traditional medicine department of 2 tertiary hospitals in Guangdong Province of China	Mean age 44.5 (range 22‐74) 76% female	“To determine Chinese patients' preferences and trade‐offs for acupuncture and low frequency infrared treatment in LBP”^72^	Quantitative: Questionnaires and interviews
Chenot (2007) 56 Germany	Acute LBP = <90 d, recurrent LBP = multiple episodes of LBP of <90 d duration within the last 12 mo, chronic LBP more than 90 consecutive days of LBP within the last 12 mo	1342	Post hoc analysis of a longitudinal study within a 3 armed RCT with an educational intervention in primary care. The RCT was to assess the impact of guideline oriented treatment on functional capacity in patients with LBP. Consecutive patients with LBP were recruited by general practitioners.	35% age <40 yo, 49% age 40‐60 yo, 22% age >60 yo 58% female	“To (i) estimate the extent of CAM use for LBP in Germany and to obtain information about the most commonly used CAM methods and (ii) to explore which disease‐related, socio‐demographic and healthcare‐related factors are associated with CAM use for LBP”^56^	Quantitative : Questionnaires and telephone interviews
Chenot (2008) 57 Germany	Acute LBP = <90 d, recurrent LBP = multiple episodes of LBP of <90 d duration within the last 12 mo, chronic LBP more than 90 consecutive days of LBP within the last 12 mo	1342	Prospective cohort study embedded within a 3‐armed RCT with an educational intervention in primary care. Consecutive patients with LBP were recruited by general practitioners.	No specialist consultation: 35% age <40 yo, 43% age 40‐60, 22% age >60 yo. 46% female. Specialist consultation: 28% age <40 yo, 47% age 40‐60, 25% age >60 yo. 54% female	“To explore (i) factors which are association with LBP patients' seeking specialist care and its appropriateness, (ii) how specialist care affects management of LBP and (iii) whether there is an over‐ and underutilization of healthcare resources”^57^	Quantitative : Questionnaires and telephone interviews
Cook (2000) 34 UK	Duration of LBP 6 mo to 21 y	7	7 patients were selected by the researcher who had attended the back rehabilitation programme in the last 6 mo	Age range 22‐53 57% female	“To explore how individual patients experienced LBP, their experience of active rehabilitation, and their perception of its influence of their subsequent ability to manage their problem”^34^	Qualitative: Semi‐structured in‐depth interviews
Cooper (2008) 35 UK	Participants who had attended at least 2 PT sessions for the treatment of chronic or recurrent non‐specific LBP and had been discharged up to 6 mo previously	25	Participants were recruited from 7 PT departments in Scotland. Purposive sampling frame was developed to ensure representation.	3: age 18‐34 8: age 35‐50 14: age 51‐65 80% female	“To define patient‐centredness from the patient's perspective in the context of physiotherapy for chronic LBP”^35^	Qualitative: Semi‐structured interviews
Cooper (2009) 36 UK	Participants who had attended ≥2 PT sessions for the treatment of chronic or recurrent non‐specific LBP and had been discharged up to 6 mo	25	Seven PT departments in Scotland. Purposive sampling frame was developed to ensure representation.	Age 18‐34: n = 3 Age 35‐50: n = 8 Age 51‐65: n = 14 80% female	“To explore the extent to which physiotherapy facilitated chronic LBP patients to self‐manage following discharge, and to explore patients' perceptions of their need for self‐management interventions or support and their preferences in terms of delivery”^36^	Qualitative: Semi‐structured interviews
Crowe (2010) 70 New Zealand	LBP >12 wk	64	Community health newsletters and physiotherapy clinics.	Mean age 55.1 (SD13.2). 48% Female	“To report on the self‐management strategies of people with chronic LBP and how their healthcare professionals perceived their role in facilitating self‐management”^70^	Qualitative: Semi‐structured interviews
Dean (2005) 37 UK	Recent exacerbation of LBP ranging from 2‐8 wk for whom the normal course of recovery from an acute episode was not apparent hence referral to primary care physiotherapy	9	Convenience sample from a local community hospital where the physiotherapist purposefully approached 9 participants from her own patient list on behalf of the researcher	Mean age 39.5 Gender distribution not specified	“To explore patients' and physiotherapists' perceptions of exercise adherence”^37^	Qualitative: Interviews
Dima (2013) 38 UK	LBP (>6 wk)	75	Participants identified from lists of patients who had recently consulted their family doctor or CAM practitioner because of LBP and members of a chronic pain patient support group.	Median age 62 (range 29‐85) 64% female	“To explore patient's beliefs about LBP treatments”^38^	Qualitative: Focus groups
Eaves (2015) 45 USA	Chronic LBP (>3 mo duration)	64	Recruited participants via advertising in clinics, yoga studios, online classified advertisements, CAM practitioners referrals of new patients, community clinic that offers CAM therapies.	Age range not specified 75% female	“To develop a valid expectancy questionnaire for use with participants starting new CAM therapies and how participants' expectations of treatment changed over the course of a therapy”^45^	Qualitative: Interviews
Ferreira (2009) 65 Australia	Duration of LBP not specified	77	Participants were recruited prior to physiotherapy intervention at a large hospital outpatient department.	Mean age 52.3 (SD 15.1) 66% female	“To assess patients' perceptions of what constitutes the smallest worthwhile effect of specific interventions for LBP”^65^	Quantitative: Interviews
Grimmer (1999) 66 Australia	Patients were included if they had been free of LBP in the previous 6‐wk period. Pain could be felt in any region of the back or leg.	21 GPs, 74 physiotherapists, 13 third party payers and 121 physiotherapy patients	General practitioners were systematically sampled from telephone books. Physiotherapists were randomly sampled from the Australian Physiotherapy Association (South Australia branch). 10 patient questionnaires were sent to each physiotherapist. All third party payer organizations with offices listed in the Adelaide metropolitan telephone book were invited to participate.	Mean age 41.8 (SD 14.3) for men and mean age 43.9 (18.2) for females 46% female.	“To compare stakeholder expectations of outcome of physiotherapy management of acute LBP”^66^	Qualitative: In‐depth interview, focus groups and questionnaires
Heyduck (2014) 58 Germany	Chronic LBP with no disc surgery within the past 6 mo	201	Study participants were recruited from 4 rehabilitation centres	Mean age 54.09 (SD 11.37) 63% female	“To (i) describe the illness and treatment beliefs of patients with chronic LBP and (ii) to explore the relation of these illness and treatment beliefs to individual, disease and interaction related variables”^58^	Quantitative: Questionnaires
Hsu (2014) 44 USA	LBP defined as having less than 2 wk without pain in the last 3 mo	64	Convenience sample of participants recruited from CAM provider offices, a research website or an online advertisement.	Average age not specified 75% female	“To provide new perspectives on the outcome expectations of patients prior to receiving CAM therapies for chronic low back pain”^44^	Qualitative: Semi‐structured interviews
Keen (1999) 39 UK	LBP >4 wk but no more than 6 mo of constant LBP for 6 mo	27	Purposive sample from individuals with low back referred by their GPs to a community‐based, single‐blind RCT to evaluate a progressive exercise programme.	Progressive exercise programme—65% female, age n = 4 18‐29 yo, n = 3 30‐29 yo, n = 3 40‐49 yo, n = 7 50‐60 yo Continue with GP advice and treatment—60% female, n = 1 18‐29 yo, n = 2 30‐39, n = 4 40‐49, n = 3 50‐60 yo	“To explore associations between factors that influence changes in physical activity and the way individuals perceive and behave with their LBP and the impact of those perceptions and recognize on physical activity”^39^	Qualitative: Interviews
Liddle (2007) 61 Ireland	Currently having or recently having LBP (non‐specific LBP) last 3 mo or more Only those who had received treatment within the previous 24 mo were included to help recognize recall bias	18	Invitation by a campus‐wide (University of Ulster) email, poster advertisement and word of mouth.	50% between with ages of 41‐55 yo 75% female	“To explore the experiences, opinions and treatment expectations in chronic LBP patients in order to identify what components of treatment they consider as being of most value”^61^	Qualitative: Focus group interviews
Lyons (2013) 51 USA	LBP >1 y	48	Recruitment by letter from patients' lists at a family medicine clinic, chiropractic academic health centre and flyers at 2 senior centres and 3 senior housing sites.	Mean age 75.2 (SD 8) 79% female	“To explore the perspectives of older adults toward LBP collaborative care by MDs (medical doctors) and DCs (doctor of chiropractic therapy)”^51^	Qualitative: Focus group interviews
May (2001) 41 UK	Duration of LBP not specified	34	Patients were recruited from 2 hospital sites in 1 town with purposive sampling of those who had received physiotherapy for LBP at some time in the previous year.	Age range 29‐77 59% female	“To describe aspects of physiotherapy care which back patients consider important”^41^	Qualitative: Interviews
May (2007) 40 UK	Duration of LBP not specified	34	Systematically sampling from a pool of patients who had received physiotherapy for LBP from two physiotherapy departments in the UK.	Age range 29‐77 59% female	“To explore patients' perspective and attitudes about back pain and it's management using an explorative qualitative approach”^40^	Qualitative: Semi‐structured interviews
Medina‐Mirapeix (2009) 63 Spain	Neck or LBP who received and finished PT in last 3 mo	34	4 public primary health‐care centres in Murcia, Spain. Mixed purposive sampling strategy was used to select participants.	Median age 48 (range 25‐70) 22/34 had neck pain 68% female	“To identify the beliefs and perceptions of patients with chronic neck and LBP that influence adherence to home exercise during exacerbation and/or remission of pain”^63^	Qualitative: Focus group interviews
Nyiendo (2000) 53 USA	Chronic LBP >6 wk	137	Participants of 45 chiropractic clinics, the outpatient clinic of the Department of Family Medicine (Oregon Health Sciences University) and 5 Portland area Family Medicine clinics.	Chiropractic patients: mean age 40.4 (13.4), 55% female Family practice patients: mean age 48.1 (15.8), 71% female	“To collect data on patient outcomes and physician practice activities in chronic recurrent LBP”^53^	Quantitative: Questionnaires
Nyiendo (2001) 52 USA	Acute and chronic LBP were enrolled (chronic is >6 wk)	835	Participants were recruited for a prospective longitudinal non‐randomized practice‐based observational study of patients self‐referring to medical and chiropractic physicians.	Not reported	“To report on long‐term pain and disability outcomes for patients with chronic LBP, evaluates predictors of long‐term outcomes and assess the influence of doctor type on clinical outcome”^52^	Quantitative: Questionnaires
Pincus (2000) 42 UK	LBP >3 mo	60	General practitioners and osteopaths recruited patients during their consultation visit.	Mean age 43 yo (SD11) 65% female	“To monitor patients' preference between 2 services offered in the same surgery for the same health problem; the traditional GPs care and a new specialist service, osteopathy”^42^	Quantitative: Interviews
Scheermesser (2012) 62 Switzerland	Chronic LBP, duration not specified. Mean duration of LBP 7 y in men and 3.5 y in women	13	Participants were purposively sampled from the Rehabilitation Centre Clinic	Mean age 52 (men) and 48 (women) 31% female 3 from Serbia, 4 from Croatia, 3 from Bosnia, 1 from Macedonia and 1 from Kosovo (living in Switzerland mean 24.5 y in men and 16 y in women)	“To identify what factors patients of Southeast European cultural background in multidisciplinary rehabilitation programs for LBP perceive to be important for acceptance or participation and are the patients' perspectives similar to those of health professionals and scientific literature?”^62^	Qualitative: Focus group and semi‐structured in‐depth interviews
Schers (2001) 64 the Netherlands	Acute LBP <6 wk Subacute 6‐12 wk Chronic >12 wk	20	Purposive sampling of 40 general practitioners from a region in the eastern Netherlands. Each GP was asked to invite the first patient of >18 yo with non‐specific LBP.	Patients median age 43 (range 25‐68) 45% female	“To explore factors that determine non‐adherence to the guidelines for LBP”^64^	Qualitative: Semi‐structured interviews
Sharma (2003) 54 USA	Duration of LBP not specified	1414	Data was derived from the baseline questionnaire of a prospective, longitudinal, non‐randomized, practice‐based observational study of patients who self‐referred to medical doctors and doctors of chiropractic therapy.	MD – age 38.7 (10.83) and 52% female. DC – age 41.5 (11.68) and 52% female	“To identify the salient determinants of patient choice between medical doctors and doctors of chiropractor for the treatment of LBP”^54^	Quantitative: Questionnaires
Sherman (2004) 11 USA	LBP >3 mo	249	Participants were recruited from a non‐profit managed health‐care system (Group Health) and a large multispecialty group practice (Harvard Vanguard).	52% of participants age <65 yo 60% female	“To determine if back pain patients are willing to try acupuncture, chiropractic, massage, mediation, tai‐chi and learn about their knowledge of, experience with and perceptions about each of these therapies”^11^	Quantitative: Questionnaire
Sherman (2010) 55 USA	Duration of LBP not specified	477	638 participants were recruited from integrated health‐care systems in the Seattle and Oakland metropolitan areas for a RCT of 4 treatments—individualized acupuncture, standardized acupuncture simulated acupuncture and usual care	Mean age 47 yo (SD13) 61% female	“To evaluate if greater improvement would be more likely in participants with; Higher baseline expectations that their back pain would improve Higher baseline expectations of the helpfulness of acupuncture A preference for acupuncture over other back pain treatments”^55^	Quantitative: Telephone interviews and short questionnaires
Sigrell (2001) 59 Sweden	LBP >2 wk duration and a history of a total of 30 d with LBP within the past year	There were 27 participants in Study 1, 17 in Study 2, 23 in Study 3, 13 in Study 4 and 20 in Study 5.	5 consecutive studies were carried out in 1 chiropractic practice where a subset of patients new to the clinic was chosen.	Mean age and gender not reported	“To design a questionnaire that can be used to identify patients' expectations of chiropractic management”^59^	Quantitative: Interview and questionnaires
Sigrell (2002) 60 Sweden	LBP of >2 wk duration and a history of more than 30 d with LBP in total within the past year	336	Chiropractic clinics in Sweden with receptionists were invited to participate in the study. Each clinic was asked to include 20 new patients.	Mean age of chiropractors 37 yo and mean age of patients 48 yo. Male 80% (chiropractors) Male:female ratio was “almost equal” in patients	“To investigate the expectations of new patients consulting a chiropractor and to evaluate differences and similarities in expectations between chiropractors and patients”^60^	Quantitative: Questionnaires
Skelton (1996) 15 UK	>1 recorded consultation for LBP	52	1 general practitioner from 12 general practices was invited to recruit up to 7 consecutive patients presenting with LBP. A maximum of 6 patients per GP were interviewed.	Median age 45 (range 31‐61) 50% female	“To explore the views of patients about LBP and its management in GP”^15^	Qualitative: Semi‐structured interviews
Slade (2009) 68 Australia	LBP >8 wk	18	Recruitment was by metropolitan and community newspaper advertisements and university email.	Mean age 51 (SD 10) 67% female	“To investigate and summarise participant experience of exercise programmes for non‐specific chronic LBP and the effects of these experiences on exercise participation and engagement”^68^	Qualitative: Focus group discussion
Slade (2009) 67 Australia	LBP >8 wk	18	Recruitment was by metropolitan and community newspaper advertisements and university email.	Mean age 51 (SD 10) 67% female	“To evaluate what factors participants in exercise programs for chronic LBP perceive to be important for engagement and participation”^67^	Qualitative: Focus group discussion
Slade (2009) 69 Australia	LBP >8 wks	18	Recruitment was by metropolitan and community newspaper advertisements and university email.	Mean age 51 (SD 10) 67% female	“To determine participant experience of exercise programs for nonspecific chronic LBP”^69^	Qualitative: Focus group discussion
Westmoreland (2007) 43 UK	Subacute or chronic neck or back pain but duration of pain not defined	20	Purposive sampling of 20 participants with subacute or chronic neck or back pain were interviewed	Age range 29‐88 75% female	“To explore patients' views of receiving osteopathy in contrast with usual GP care, to provide insight into the psychological benefit of treatment, and to explore their views on how such a service should be provided and funded”^43^	Qualitative: Semi‐structured interviews preceded by short questionnaires
Yardley (2010) 32 UK	LBP >3 mo	383	Participants recruited from 64 general practices in south and west of England. For the interview study, participants were purposively recruited from each intervention by phone.	Age and gender distribution not specified for questionnaire study. Age range 31‐61 for interview study; 54% female	“To understand trial participants' expectations and experiences of the Alexander Technique and exercise prescription”^32^	Mixed‐methods: Interviews and questionnaires

The duration of LBP was either undefined or mixed in 36 (82%) studies,^14^, ^15^, ^35^, ^36^, ^37^, ^38^, ^39^, ^40^, ^41^, ^43^, ^44^, ^47^, ^48^, ^49^, ^50^, ^52^, ^53^, ^54^, ^55^, ^56^, ^57^, ^58^, ^59^, ^60^, ^61^, ^62^, ^63^, ^64^, ^65^, ^66^, ^67^, ^68^, ^69^, ^71^, ^72^ 8 (18%) studies reported on chronic LBP(>12 weeks duration).^11^, ^32^, ^33^, ^42^, ^45^, ^46^, ^51^ There were no studies on acute LBP (<4 weeks duration). The median age of participants was 47 years of age with a female predominance (62% females). There were 25 qualitative,^15^, ^17^, ^33^, ^34^, ^35^, ^36^, ^37^, ^38^, ^39^, ^40^, ^41^, ^43^, ^44^, ^45^, ^46^, ^51^, ^61^, ^62^, ^63^, ^66^, ^67^, ^68^, ^69^, ^70^, ^71^ 18 quantitative^11^, ^14^, ^42^, ^48^, ^49^, ^50^, ^52^, ^53^, ^54^, ^55^, ^56^, ^57^, ^58^, ^59^, ^60^, ^65^, ^72^ and 1 mixed‐methods study.^32^ The median number of participants in the qualitative studies was 23 (range 7‐121), and the median number of participants in quantitative studies was 643 (range 60‐1555).

### 3.2 Quality of studies

Quality assessments of the included studies are presented in the Appendix S1: Figures S1 and S2. Of the qualitative studies, physiotherapy care was examined in 18 studies, CAM in 11 studies and chiropractic therapy in 2 studies. Across all disciplines, the quality assessments for qualitative studies reflected potential biases in data collection and of the studies examining physiotherapy and CAM, recruitment strategies were also at risk of bias. The quantitative studies were of low quality. Of the 18 quantitative studies, the 5 studies that examined physiotherapy care were all at high risk of bias. Seven quantitative studies examined CAM, with 6 studies being at high risk and 1 study of moderate risk of bias. Of the 8 studies that examined chiropractic therapy, 4 were at moderate risk of bias and 4 were at high risk of bias.

### 3.3 Results of review

Three main areas of perceived need emerged (Table 2).

**Table 2:** Patients perceived needs of allied health and complementary and alternative medicine (CAM) related to back pain

Author & Year	Results
**Physiotherapy and exercise therapy (includes therapeutic exercise, general exercise or physical activity guided or prescribed by a physiotherapist; manual therapies; education; other physical therapies or aids**)	
Expectations for physiotherapy, including a preference for physiotherapy and exercise therapy	
Amonkar (2011)^14^	Patients value physiotherapy/osteopathy more than care delivered by medical practitioners
Cooper (2009)^36^	All participants “wanted direct access to a physiotherapist” and/or “follow up in the future”^36^ The physiotherapist was seen as the expert in LBP Some participants thought that is “would be helpful if the patient was able to telephone the physiotherapist, using it as a form of helpline for LBP”^36^
Crowe (2010)^70^	“Most participants recognized exercise as effective”^70^ “Low impact exercise was strongly favoured as a self‐management strategy by participants”^70^ 15 participants identified that “healthcare professionals played a role in their self‐management”^70^ “The nominated professionals were predominantly physiotherapists or general practitioners”^70^
Ferreira (2009)^65^	On average, “patients perceived that an intervention would have to make them ‘much better', which corresponded to 1.7 (SD 0.7) on the 4 point scale or improve their symptoms by 42% to make it worthwhile”^65^
Grimmer (1999)^66^	Patients chose to attend their physiotherapist for a variety of reasons, the most common of which were “convenience, reputation, previous good experience and/or recommendation”^66^
Liddle (2007)^61^	Participants clearly recognized the value of exercise
May (2001)^41^	Patients expected physiotherapist‐delivered and discussion about personal worries as management of their back pain
May (2007)^40^	Participants found exercises an “important part of the management of their problem”^40^
Medina‐Mirapeix (2009)^63^	Only a few “patients prefer continual exercise, most prefer exercising only if pain reappears”^63^
Schers (2001)^64^	Only a few patients “would ask for a referral to a physiotherapist when symptoms would last a few more weeks”^64^ Patients thought of physiotherapy “mainly as massage or other passive treatment”^64^
Yardley (2010)^32^	Exercise therapy and the Alexander Technique were perceived to be unlikely to cause harm, therefore participants were willing to try these interventions even when expectations for benefit were felt to be minimal Exercise therapy and the Alexander Technique were perceived to be another “opportunity to try something new since previous attempts to relieve back pain were unsuccessful”^32^
Beliefs about physiotherapy and exercise	
Dima (2013)^38^	Patients believe that manual therapies realign the spine, release the nerves and strengthen the muscles. They feel that physiotherapy results in temporary relief, and maintains/prevents worsening and cures back pain. Patients perceive exercise as strengthening muscles, reducing stiffness, improves mental state and weight loss. They think exercise results in temporary relief, maintenance, enables activity and cure.
Grimmer (1999)^66^	“Patients expected symptom relief at the end of the first treatment”^66^
Heyduck (2014)^58^	“Patients had very high expectations about rehabilitation (i.e. that it addresses their personal needs and is diversified)”^58^ They had high expectations on the results of rehabilitation, that is that it improves somatic and psychological aspects.
Medina‐Mirapeix (2009)^63^	“Patients believe that continuing exercises might prevent relapse but they face a conflict between knowing that they should perform and feeling it is difficult to adhere”^63^
Scheermesser (2012)^62^	9 of 13 agreed that “activity has a positive impact on health”; however, the “majority of patients felt that exercise was good but did not improve back pain”^62^
Slade (2009)^67^	“All participants acknowledged the importance of an exercise environment based on health promotion rather than remediation of the sick/injured”^67^ 12 of 18 participants reported that gym equipment was useful
Yardley (2010)^32^	“Few participants hope for a complete cure, but many were desperate to attain some degree of pain relief”^32^ “Patients wanted insight into how to prevent or manage episodes of back pain better”^32^ Exercise therapy and the Alexander Technique were perceived to be unlikely to cause harm, therefore participants were willing to try these interventions even when expectations for benefit were felt to be minimal
Individualizing physiotherapy and exercise	
Keen (1999)^39^	“Health professionals were rarely effective in enabling a participant to sustain (6 + months) increased physical activity except where an individual had regular contact with a health professional”^39^ Exercise advice needs to be “tailored to the individual's circumstance”^39^
Liddle (2007)^61^	Patients “need individual exercises and advice regarding suitable lifestyle adaptations”^61^ “Supervision of exercise programmes was considered important to provide individual correction”^61^ “Participants wanted follow up and reassurance from the practitioner that they were carrying out instructions correctly and assistance with appropriate treatment progression in line with their stage of recovery”^61^
Medina‐Mirapeix (2009)^63^	Patients know that they should perform exercises; however, they find it difficult to adhere
Slade (2009)^67^	“All participants reported that they developed preferred exercise styles over time. The range of preferred exercise styles reinforced that the individual should be consulted in program design”^67^ “Preferences ranged from individual to group, from unsupervised to closely supervised and included minimal disruption to their lives and exercises as part of recreational or routine daily practices”^67^
Yardley (2010)^32^	Patients “valued hands‐on care, emotional support and detailed advice provided”^32^
Concerns with physiotherapy and exercise	
Dima (2013)^38^	They are concerned that it feels sore after manipulation, causing further damage and ‘cracking' bones. They are concerned about injuring the back and have difficulties maintaining motivation.
Slade (2009)^67^	6/18 participants thought that “gyms were intimidating and prevented them from exercise engagement”^67^ All “reported that compliance was difficult when they lacked confidence in correct exercise performance”^67^
Westmoreland (2007)^43^	“Disadvantages included the lack of a specific diagnosis, ineffective treatment and long waiting lists”^43^
Yardley (2010)^32^	Some participants were concerned that exercise therapy would make pain worse from previous experience Some patients were “concerned that physiotherapy would be difficult to fit into their lifestyle”^32^ “Free time, bad weather, cost and lack of social support were perceived as obstacles to engaging in physiotherapy”^32^ Exercise therapy was often perceived as unpleasant or difficult to keep up
**Chiropractic therapy**	
Willingness to try chiropractic therapy or preference for chiropractic therapy	
Carey (1996)^48^	“61% of adults with acute severe LBP did not seek any health care during their most recent episode of pain however 24% initially sought care from a physician, 13% from a chiropractor and 2% sought care from other providers (physical therapist, nurse, massage therapist)”^48^
Lyons (2013)^51^	Participants across groups considered “chiropractic a primary not complementary LBP treatment and said that DCs offered many modalities”^51^
Perceived benefit, expectations and concerns with chiropractic therapy	
Borkan (1995)^71^	“Non orthodox and folk healers (include reflexology, chiropractors, acupuncture, spiritual healers, movement therapy) often perceived as being more empathic, more knowledgeable and having better diagnostic skills and providing more effective therapies”^71^
Carey (1995)^49^	“Patients who saw chiropractors reported a significantly higher degree of satisfaction than those who saw practitioners” (primary care physicians, orthopaedics and HMO) in the other 4 strata.^49^ “Higher level of satisfaction among the patients who saw chiropractors persisted after adjustment for the number of visits and the use of radiography”^49^
Carey (1996)^48^	“Those who sought care from chiropractors were more likely to feel that treatment was helpful (99% vs 80%, p = 0.001) and less likely to seek care from another provider for that same episode of pain (14% vs 27%)”^48^
Lyons (2013)^51^	Patients “expected chiropractors to provide hands on treatments or spinal manipulation to deal with the cause of the pain”^51^ “Some participants noted that chiropractic adjustments did not relieve their LBP for several treatments, provided short term relief or produced side effects e.g. muscle pain”^51^
Nyiendo (2001)^52^	More participants reported satisfaction in the chiropractic group compared to patients treated by family physicians
Nyiendo (2001)^53^	“Satisfaction was higher for patients attending chiropractors (compared to physicians)”^53^ “Chiropractic patients expressed greater satisfaction regarding information and treatment provided”^53^ “Chiropractic patients also reported greater improvement at 1 mo as measured by subjective assessment”^53^
Sigrell (2001)^59^	“Patients' main expectations of chiropractic management are an accurate diagnosis, an explanation of the complaint or affliction and treatment that results in a positive outcome”^59^
Sigrell (2002)^60^	“High agreement on the expectations that the chiropractors should find the problem and should explain the problem to the patient”^60^ “Agreement that the patient should feel better and be free of symptoms”^60^ “80% of patients agreed that ‘the patient' should be given advice about training and exercises”^60^
Characteristics of patients preferring chiropractic therapy	
Carey (1996)^48^	Chiropractic care was more common among men than women and among younger adults than older “Those whose acute episode of pain did not begin at work were more likely to seek chiropractic care (66% vs 43%, *P* = .05)”^48^ “Employed individuals were more likely to seek care from chiropractors”^48^ No association between seeking care from chiropractor vs medical doctor based on ethnicity, education, income, insurance, worker's compensation status, population density, perceived health status, presence of leg pain or a previous history of surgery or recognition for LBP Strongest independent predictors of seeking care from a chiropractor for acute LBP was male gender, age <60 yo, attribution of cause to back pain to disc disease
Carey (1999)^50^	Proportion of chiropractic patients seeking care is greater than the proportion of patients with functionally disabling symptoms
Sharma (2003)^54^	“Self‐referral to chiropractors was associated with history of LBP and acute LBP”^54^ High proportion of self‐pay patients with chiropractors “Older and higher income patients were more likely to select chiropractors”^54^ “Patients who expressed confidence in the ability of their chosen providers to successfully treat their LBP were more likely to obtain care from chiropractors than were patients who lacked such confidence (OR6.08, 95%CI 3.84‐9.63)”^54^ “Patients with more favourable attitudes toward self‐directed treatment and active behavioural involvement were somewhat more likely to choose chiropractors (OR 1.13, 95% CI 1.06‐1.21)”^54^ “Patients who were opposed to prescription drugs were more likely to choose chiropractors (OR 1.57, 95% CI 1.11‐2.22)”^54^
**Complementary and alternative therapies for lbp (including acupuncture, osteopathy, massage therapy, local heat therapy**)	
Willingness to try CAM or preference for CAM	
Allegretti (2010)^46^	Patients were generally willing to try” “complementary and alternate therapies”
Astin (1998)^47^	“4.4% of patients reported relying primarily on alternate therapies”^47^
Chen (2015)^72^	Patients of female gender were less willing to receive either acupuncture or low frequency infrared treatment Higher out‐of‐pocket costs decreased patients' willingness to try these therapies Patients expected major pain reduction by 12 courses of therapy
Chenot (2007)^56^	“A large proportion of patients with back pain are using at least 1 form of CAM, mostly in the form of local heat, massage, and spinal manipulation”^56^
Scheermesser (2012)^62^	“Almost all interviewed patients prefer Western medical treatment over traditional treatment”^62^
Sherman (2004)^11^	“More than half the respondents said they would be very likely to try acupuncture, chiropractic therapy or massage provided by their health plan for no additional cost and if their physician felt it was reasonable”^11^ “Fewer respondents said they would be very likely to try meditation or Tai‐chi”^11^ “Respondents believed that massage would be most helpful CAM therapy for their current back pain and that meditation would be least helpful CAM”^11^
Sherman (2010)^55^	At baseline 1/3^rd^ of participants wanted acupuncture
Skelton (1996)^15^	“Of 37 patients who had never used CAM, 13 were largely satisfied with the care they were receiving and not considered an alternative and 6 had never heard of any form of CAM”^15^ 10 of 52 patients had consulted CAM (mainly osteopaths and chiropractors) and most of these “patients thought of CAM as experimental or as a desperate measure when their pain became intolerable or when an immediate GP consultation was unavailable or likely to be ineffective”^15^
Westmoreland (2007)^43^	“General agreement that NHS should provide spinal manipulation”^43^
Perceived benefit of CAM and satisfaction with CAM	
Astin (1998)^47^	“2 most frequently endorsed benefits from CAM were ‘I get relief for my symptoms, the pain or discomfort is less or goes away, I feel better'”^47^
Borkan (1995)^71^	“Non orthodox and folk healers (include reflexology, chiropractor, acupuncture, spiritual healers, movement therapy) often perceived as being more empathic, more knowledgeable and having better diagnostic skills and providing more effective therapies”^71^
Crowe (2010)^70^	Some participants found heat therapy effective
Dima (2013)^38^	Patients think that acupuncture stimulates nerves, relaxes muscles and results in temporary relief or cure.
Eaves (2015)^45^	Some patients view engagement with CAM as a means to help them accept the personal responsibility for managing pain and contribute to positive behaviour change
Hsu (2014)^44^	Patients hoped that CAM would reduce pain, however many expected the amount of pain relief to be modest Many participants wanted CAM to help with functional outcomes, in particular “increase the ability to do activities which focused on work, hobbies, social life and activities of daily living”^44^ Some participants expected CAM “to improve physical fitness, in particular muscle strength, flexibility and overall fitness”^44^
May (2007)^40^	Participants found heat and massage therapy helpful, as well as wearing a corset at work
Pincus (2000)^42^	There was higher satisfaction with osteopathy than GPs in the practice “The difference was stronger for aspects of care/communication and competence with osteopathy and weaker for satisfaction and efficacy”^42^
Westmoreland (2007)^43^	“Osteopathy was though to have reasonable premise as it involved moving or manipulating joints, which were loosened and put back into place”^43^ “Physical benefits of osteopathy included pain relief, feeling better, looser with relief of tension and increased mobility”^43^ “Psychological benefits included reassurance and improved understanding”^43^ “Osteopathy also included the removal of fear and positive approach, which encouraged exercise rather than rest”^43^ “Longer consultations with osteopathy allowed more time for explanation and thorough physical examination developed good rapport”^43^
Concerns with CAM	
Campbell (2007)^33^	Despite endorsements for “complementary and alternate therapies,” the treatments were “viewed as having only transitory effects and unlikely to be maintained especially when participants had to personally bear the burden of the treatment costs”^33^ “Appeared to recognize that the therapies allied to medicine (including osteopathy and reflexology) were limited in terms of the relief that they provided because the treatments were perceived to stand outside of the medical model”^33^
Dima (2013)^38^	Patients are concerned about painful needling, fear of needles with acupuncture
Eaves (2015)^45^	Despite initial improvement in pain, patients reported disappointment that massage therapy did not offer a cure
Skelton (1996)^15^	Of 37 patients who had never used complementary and alternate therapies, “8 questioned its legitimacy and feared being ripped off, 10 were unable to purse CAM through lack of information or lack of money”^15^
Westmoreland (2007)^43^	Adverse psychological effects of spinal manipulation included “that it was surprising, unexpected, initially frightening and embarrassing”^43^

### 3.4 Patients' perceived needs related to the use of physiotherapy (incorporating therapeutic exercise, general exercise or physical activity guided or prescribed by a physiotherapist, manual therapies, education, other physical therapies or aids commonly applied or used by a physiotherapist)

#### 3.4.1 Extent of need for physiotherapy

Eleven studies identified patients' preference for and willingness to try physiotherapy.^14^, ^32^, ^36^, ^40^, ^41^, ^61^, ^63^, ^64^, ^65^, ^66^, ^70^ Participants reported the benefits of exercise and some valued physiotherapist‐delivered care more than care delivered by a medical practitioner.^14^, ^32^, ^61^, ^66^, ^70^ Many studies reported that patients thought exercise was an important component of care for LBP and expected physiotherapist‐delivered care in this context, incorporating therapeutic exercise and discussion about the impact of their LBP experience, as part of their LBP management.^40^, ^41^ However, some patients would ask for a referral to a physiotherapist only when symptoms lasted for at least a few weeks.^64^ Some patients preferred exercising only when pain reappeared rather than continual exercise.^63^ Cooper reported that patients wanted direct access to physiotherapists and some patients thought that it would be helpful if they were able to telephone the physiotherapist, using it as a form of helpline for LBP management.^36^

#### 3.4.2 Perceived benefit of physiotherapy care

Seven papers identified the patients' perceived benefits of physiotherapy.^32^, ^38^, ^58^, ^62^, ^63^, ^66^, ^67^ Of these studies, the main themes that emerged were that physiotherapy resulted in temporary relief of pain,^32^, ^38^, ^43^, ^66^ prevented worsening of LBP^32^, ^38^, ^63^ and helped with mobility and function.^38^ Patients wanted to learn pain management strategies through physiotherapy care,^32^ and Grimmer reported that patients expected symptom relief at the end of the first treatment.^66^ Physiotherapy was also perceived as being helpful for injuries, muscle strengthening, reducing stiffness, “realigning the spine” and “releasing the nerves”.^38^, ^43^ Furthermore, patients believed that physiotherapy fostered health promotion,^67^ addressed their personal needs,^58^ improved their mental state^38^, ^58^ and helped with weight loss.^38^ Yardley found that participants believed there would be little harm from physiotherapy care.^32^

#### 3.4.3 Individualizing physiotherapy care

Five studies reported patients' preference for individualizing physiotherapy care, tailored to the patients' perceived needs.^32^, ^39^, ^61^, ^63^, ^67^ Patients desired advice regarding suitable lifestyle adaptations and physiotherapy interventions tailored to their individual health needs, particularly within the context of exercise prescription.^39^, ^61^, ^67^ Slade found that some participants preferred group exercise programmes, while others desired an individual exercise regimen, thus highlighting the importance of considering patient preference in designing an exercise programme tailored to meet their specific needs.^67^ They also felt that supervision and follow‐up of their exercise programme were important,^61^ and that without regular contact, health professionals were rarely effective in supporting participants to continue increased physical activity.^39^ Furthermore, patients wanted reassurance from the practitioner that they were performing the exercises correctly and other self‐management strategies.^61^

#### 3.4.4 Concerns with physiotherapy care

Patients' concerns related to physiotherapy were explored in 5 studies.^32^, ^38^, ^43^, ^63^, ^67^ Dima reported that patients were afraid of injuring their back with physiotherapy.^32^, ^38^ They reported feeling sore after manipulation, which they believed may cause further damage to their back.^38^ Moreover, patients were concerned about their ability to adhere to an exercise programme, especially due to a lack of free time, cost and lack of social support.^32^, ^63^ They also lacked confidence in correct exercise technique, which affected their compliance with rehabilitation.^67^ Furthermore, patients were concerned about the lack of a specific diagnosis given by physiotherapists and that the treatments were ineffective.^43^

### 3.5 Patients' perceived needs for chiropractic therapy

#### 3.5.1 Willingness to try chiropractic therapy

Two studies reported on patients' willingness to try chiropractic therapy.^48^, ^51^ Lyons' study found that participants recruited from chiropractic and general practice clinics considered chiropractors as primary therapists rather than complementary therapists for LBP.^51^ Carey reported that 13% of adults with acute severe LBP sought care from a chiropractor.^48^

#### 3.5.2 Perceived benefit, expectations and concerns with chiropractic therapy

Eight studies described patients' perceived benefit, satisfaction and expectations of chiropractic therapy.^48^, ^49^, ^51^, ^52^, ^59^, ^60^, ^71^ Three studies reported that patients who consulted chiropractors were satisfied with their management.^49^, ^52^, ^53^ According to Sigrell's results, patients expected chiropractors to provide an accurate diagnosis and explain the cause of pain,^59^ as well as offer advice about training and exercises.^60^ Patients also expected that they should feel better and be free of symptoms with chiropractic therapy^60^ and they wanted hands‐on treatment or spinal manipulation from their chiropractors to treat the cause of pain.^51^ One study explored the patients' concerns with chiropractic therapy.^51^ Lyons concluded that some patients found chiropractic adjustments to not relieve their LBP for several treatments or that it provided short‐term relief and produced side‐effects such as muscle pain.^51^

#### 3.5.3 Characteristics of patients preferring chiropractic therapy

Three studies explored the characteristics of patients who preferred chiropractic therapy.^48^, ^50^, ^54^ Chiropractic care was more commonly preferred by males, employed individuals, those with more functionally disabling pain and those of higher income or self‐funded individuals.^48^, ^50^, ^54^ Other characteristics of patients preferring chiropractic care included those with more favourable attitudes towards self‐directed treatment and active behavioural involvement, patients who were more opposed to prescription medications, patients who expressed confidence in the ability of their chosen health provider, those with acute LBP, patients with LBP that did not begin at work and patients who attributed the cause of their LBP to disc disease.^48^, ^54^ There were conflicting conclusions about the age range of patients preferring chiropractic therapy.^48^, ^54^

### 3.6 Patients' perceived needs for CAM

#### 3.6.1 Degree of need for CAM

Nine studies reported patients' willingness to try CAM, with mixed results.^11^, ^15^, ^43^, ^46^, ^47^, ^55^, ^56^, ^62^, ^72^ Astin reported that 4.4% of patients relied primarily on CAM.^47^ Four studies found that patients were willing to try CAM, mostly in the form of acupuncture, massage therapy, spinal manipulation and local heat therapy.^11^, ^43^, ^46^, ^56^ Also, Sherman found in a population of patients with chronic non‐specific LBP recruited from integrated health systems to participate in a study comparing acupuncture and usual care, that one‐third of participants at baseline wanted acupuncture.^55^ However, Scheermesser reported in a study of patients with chronic LBP recruited from a Rehabilitation Centre Clinic prefer Western medical treatment to CAM. Skelton found that primary care patients viewed CAM as experimental or a desperate measure when their pain became intolerable or when medical doctors were unavailable for consultation.^15^ One study by Chen found that higher out‐of‐pocket costs incurred by acupuncture or low frequency infrared radiation treatment, as well as female gender, were associated with less willingness to try these therapies.^72^

#### 3.6.2 Perceived benefit and satisfaction with CAM

There were 9 studies that explored patients' satisfaction with CAM and the perceived benefit of CAM.^38^, ^40^, ^42^, ^43^, ^44^, ^45^, ^47^, ^70^, ^71^ Patients felt that CAM could address physical impairments perceived to be the cause of LBP, specifically CAM could relax muscles, stimulate nerves, manipulate and loosen joints and provide pain relief.^38^, ^43^, ^44^, ^47^ They also sought CAM therapies to improve function and physical fitness.^44^ May and Crowe reported that some patients felt that heat therapy^70^, ^73^ and massage therapy were effective.^40^ Patients thought the CAM practitioners were more empathic and understanding and had better diagnostic skills compared to medical doctors.^42^, ^43^, ^71^ CAM practitioners were also perceived to provide longer consultations that allowed more time for thorough examination and explanation of the diagnosis.^43^ Furthermore, patients thought there were psychological benefits of CAM, including reassurance, removal of fear and a positive approach.^43^, ^45^

#### 3.6.3 Concerns with CAM

Patients' concerns with CAM were addressed in 5 studies.^15^, ^33^, ^38^, ^43^, ^45^ Patients commented on the fear of needling and pain from acupuncture.^38^ Westmoreland reported on patients' apprehensions with adverse psychological effects of spinal manipulation including fright and embarrassment.^43^ Furthermore, some patients believed that CAM therapies provided limited and transient effects, which were perceived to stand outside of the biomedical model, and Skelton reported that some patients questioned its legitimacy and feared being “ripped off”.^15^, ^33^

## 4 DISCUSSION

This review identified 44 relevant articles reporting patients' perceived needs for physiotherapy and chiropractic care, and CAM therapy for LBP. Patients with LBP perceived a role for physiotherapy and chiropractic therapy but were concerned about adherence to treatment, correct exercise technique and adverse outcomes. The perceived needs for CAM were inconsistent, based on concerns about efficacy and adverse effects.

These findings may assist in informing the development of patient‐centred guidelines for LBP that build on the alignment of patients' perceived needs with evidence‐based management strategies, thus increasing evidence translation and appropriate health‐care provision, which in turn will assist in improving outcomes for both patients and the health‐care system.

Physiotherapy was viewed as important by patients. Patients believed that physiotherapy‐delivered care helped with pain relief, facilitated better understanding of pain management strategies, prevented worsening of LBP and improved mobility and function.^38^, ^58^, ^63^, ^66^ These patient beliefs are aligned with current clinical practice guidelines, which are based on moderate evidence supporting the role of active rehabilitation strategies such as exercise for LBP.^74^ Furthermore, a common theme that emerged from this review was the patients' desire for physiotherapist‐delivered care to be individually tailored to their health needs and abilities,^39^, ^61^, ^67^ particularly in the context of exercise prescription. Importantly, tailored care underpins a patient‐centred approach to care delivery within a biopsychosocial framework. While subgrouping patients with non‐specific LBP based on physical or psychological profiles may have benefit, definitive conclusions about the validity and efficacy of this approach in physiotherapy practice remains uncertain.^75^ Nonetheless, emerging evidence and models of care support a tailoring approach that integrates physical and psychological factors in care delivery for people with chronic LBP,^76^, ^77^ as well as matching components of care based on prognostic risk factors for recovery.^78^ Despite these advances in knowledge, practitioners encounter challenges in translating such evidence into practice, which may account for variance in outcomes.^79^, ^80^ Patients were also concerned about adherence to, and competence with, exercise programmes and the potential for further damage to their back with physical and exercise therapies. These concerns, and potentially unhelpful beliefs around persistent pain and tissue damage, may stem from a perceived lack of information received by patients regarding the pathogenesis of LBP, best‐practice care in the context of physical therapies and confusion about correct techniques for exercise therapy.^81^ As these present potential barriers to active and sustained patient involvement in active rehabilitation, particularly exercise, health‐care providers need to educate patients about the role of appropriate physiotherapy and exercise therapy, particularly regarding its safety, in order to promote confidence and sustained self‐management. Furthermore, the mode of delivery of physiotherapy and effective communication should be examined: future research targeted to assess the efficacy of alternative models of service delivery for physiotherapist‐directed care.

Chiropractic therapy was perceived by some patients to be effective; however, others were concerned about adverse outcomes. The evidence for chiropractic therapy for LBP management is controversial.^82^, ^83^ This review found that patients had conflicting views regarding chiropractic therapy for the management of LBP. While patients preferred chiropractors to medical practitioners in the management of their LBP,^48^, ^49^, ^52^, ^53^ there were no studies comparing patient preference for chiropractic therapy versus physiotherapy. Some patients were satisfied with chiropractic therapy.^49^, ^51^, ^52^, ^53^, ^60^ However, other patients felt that it only provided temporary relief and produced side‐effects such as muscle pain.^49^, ^51^, ^60^ Chiropractic care was more commonly preferred by males, those in employment, those with higher incomes and patients with more favourable attitudes towards self‐directed treatment and those opposed to prescription medications.^49^, ^60^ Given that chiropractic therapy is widely used, driven by patients' desire for rapid non‐pharmacological pain relief, and a belief that the experience of LBP is attributable to a structural cause amendable to chiropractic therapy, there is a need for further evaluation of the efficacy of chiropractic therapy for LBP.

Despite the very limited evidence to support the use of CAM for LBP,^8^, ^84^ over one‐third of patients with LBP report using CAM.^85^ This review found that patients believe that these therapies provide pain relief, loosen muscles and stimulate nerves^38^, ^40^, ^43^, ^44^, ^47^, ^70^, ^73^ and they sought CAM to improve function and physical fitness.^44^ These perceptions again highlight the attribution of LBP to a structural cause. Some patients also perceived CAM practitioners to be more understanding, empathetic and provide more time for consultations than medical practitioners and more capable of providing a diagnosis.^38^, ^41^, ^42^, ^43^, ^47^, ^70^, ^71^ However, other patients questioned the legitimacy of CAM and felt that it offered only transitory effects.^15^, ^33^, ^38^, ^43^, ^45^ The prevalence of CAM use in people with LBP is almost double that of the general population, and more than for other chronic conditions such as arthritis. This may reflect the limited efficacy of conventional treatments for LBP^86^, ^87^, ^88^, ^89^, ^90^, ^91^, ^92^ and the importance of pain relief for the management of LBP.^33^, ^46^, ^66^ It may also reflect patients' preference for spending time with health‐care providers^41^, ^93^ and their desire for a holistic approach to LBP management^94^ coupled with a meaningful diagnosis. Furthermore, patients expressed dissatisfaction with the poor communication around the aetiology of LBP and the limited therapeutic options provided by conventional health‐care practitioners.^3^, ^95^, ^96^ Thus, the higher utilization of CAM for LBP may reflect the patients' need to seek a diagnosis for their pain.^35^, ^46^, ^61^, ^94^, ^97^, ^98^, ^99^, ^100^, ^101^, ^102^, ^103^, ^104^ This highlights a need to educate patients about the mechanism of LBP and its natural history from a contemporary pain biology perspective. Despite the widespread use of CAM, the current evidence supporting the use of these therapies is limited.^8^, ^105^ Given the need and utilization of CAM, further studies are required to improve our understanding of the role of CAM, the evidence base for their use and potential for harm, thus guiding more cost‐effective utilization of health‐care resources.

## 5 LIMITATIONS AND STRENGTHS

This review has a number of limitations. First, there have been few studies that directly examined the patients' perceived need for allied health and CAM. Thus, areas of perceived need have been determined from studies that are heterogeneous in their aims and designs, mainly conducted in English‐speaking countries, had small sample sizes and were susceptible to bias. This may affect the ability of included studies to capture all areas of perceived need, and further research is required to further explore the patients' perceived need for allied health and CAM. The quality of the studies included in this review tended to be of low or medium quality, reflecting potential biases with recruitment strategy and data collection. Another limitation of this review is that there were no articles that examined participants with acute LBP only; therefore the results cannot be extrapolated to those with acute presentations of LBP. Furthermore, many of the included studies did not provide information regarding comorbidities, the severity of LBP and use of combination therapies. These factors may influence the patients' perception of need for services and health care. For example, pain‐related disability (and to some extent pain severity) is directly related to care‐seeking behaviour.^106^ Reporting these relevant descriptive factors may be an important feature of core reporting criteria for epidemiologic research in health services research related to LBP. Moreover, the majority of included articles evaluated middle‐aged participants, with few articles focusing on younger and older populations, where LBP is also prevalent and represents an important cause of disability.^3^, ^107^ Additionally, some of the included studies are over 10 years old, and so care is needed in extrapolating these data to current patient needs for CAM and allied health. Despite these limitations, this review provides a comprehensive overview of the existing literature from 4 databases and included both qualitative and quantitative methodologies. Moreover, many of the findings were consistent across several studies, reflecting the strength of the findings.

## 6 CONCLUSIONS

Our results suggest that patients may need more evidence‐informed information about the mechanisms of LBP and its natural history and the effectiveness of current therapies for LBP.^108^ Indeed, this aligns with similar calls in other conditions, such as osteoarthritis.^109^ An improved societal understanding and health literacy related to LBP may reduce barriers to uptake of evidence‐based care, such as active rehabilitation strategies.^110^ Further, improved community understanding of LBP may better facilitate more effective provider‐patient relationships. Given the patient beliefs related to services without a strong evidence base, such as CAM and chiropractic therapies, there is a need to examine these interventions to determine efficacy or better communicate more effective care pathways to patients. Here, meaningfully involving patients and/or consumer organizations in the development and dissemination of clinical practice guidelines may better align their expectations for health care with evidence.^111^

There is an increasing emphasis on patient‐orientated care for chronic conditions such as LBP. The patient's perception of need drives their use of health services. Despite the evidence for active therapies for LBP, adherence is low and mirrors other chronic musculoskeletal pain conditions, like osteoarthritis.^112^ This review has shown that this may be because current therapies do not meet patient's expectations regarding a need for holistic personalized care, pain control or an explanation for their symptoms. Thus, they turn to other modalities of care. These perceived gaps in conventional care need to be addressed and incorporated into usual practice by health‐care practitioners. Future initiatives to educate patients regarding the mechanism of LBP, its natural history and the effect of current therapies are required. Furthermore, research into developing more effective pain management strategies, improved communication about pain biology particularly as it relates to chronic non‐specific LBP and filling the evidence gaps around the efficacy and appropriate use of chiropractic therapy and CAM modalities used by patients are urgently needed.^84^ These may enable the incorporation of patient perceived needs into the recommended management of LBP to improve outcomes.

## AUTHOR CONTRIBUTIONS

All authors including Louisa Chou, Tom Ranger, Waruna Peiris, Flavia Cicuttini, Donna Urquhart, Andrew Briggs and Anita Wluka made substantial contributions to the conception and design of the study, the analysis and interpretation of the data, drafting and revision of the article and final approval of the version to be submitted. Anita Wluka (anita.wluka@monash.edu) and Louisa Chou (louisa.chou@monash.edu) take responsibility for the integrity of the work as a whole.

## References

[1] Hoy D , March L , Brooks P , et al. The global burden of low back pain: estimates from the Global Burden of Disease 2010 study. Ann Rheum Dis. 2014;73:968‐974. 24665116 10.1136/annrheumdis-2013-204428

[2] Rubin DI . Epidemiology and risk factors for spine pain. Neurol Clin. 2007;25:353‐371. 17445733 10.1016/j.ncl.2007.01.004

[3] Hoy D , Brooks P , Blyth F , Buchbinder R . The Epidemiology of low back pain. Best Pract Res Clin Rheumatol. 2010;24:769‐781. 21665125 10.1016/j.berh.2010.10.002

[4] Katz JN . Lumbar disc disorders and low‐back pain: socioeconomic factors and consequences. J Bone Joint Surg. 2006;88(suppl 2):21‐24. 16595438 10.2106/JBJS.E.01273

[5] Dagenais S , Tricco AC , Haldeman S . Synthesis of recommendations for the assessment and management of low back pain from recent clinical practice guidelines. Spine J. 2010;10:514‐529. 20494814 10.1016/j.spinee.2010.03.032

[6] Pillastrini P , Gardenghi I , Bonetti F , et al. An updated overview of clinical guidelines for chronic low back pain management in primary care. Joint Bone Spine. 2012;79:176‐185. 21565540 10.1016/j.jbspin.2011.03.019

[7] Koes BW , van Tulder M , Lin CW , Macedo LG , McAuley J , Maher C . An updated overview of clinical guidelines for the management of non‐specific low back pain in primary care. Eur Spine J. 2010;19:2075‐2094. 20602122 10.1007/s00586-010-1502-y PMC2997201

[8] van Tulder MW , Furlan AD , Gagnier JJ . Complementary and alternative therapies for low back pain. Best Pract Res Clin Rheumatol. 2005;19:639‐654. 15949781 10.1016/j.berh.2005.03.006

[9] Zollman C , Vickers A . What is complementary medicine? BMJ. 1999;319:693. 10480829 10.1136/bmj.319.7211.693 PMC1116545

[10] Eisenberg DM , Davis RB , Ettner SL , et al. Trends in alternative medicine use in the united states, 1990‐1997: Results of a follow‐up national survey. JAMA. 1998;280:1569‐1575. 9820257 10.1001/jama.280.18.1569

[11] Sherman KJ , Cherkin DC , Connelly MT , et al. Complementary and alternative medical therapies for chronic low back pain: what treatments are patients willing to try? BMC Altern Med. 2004;4:9. 10.1186/1472-6882-4-9 PMC503394 15260884

[12] Majumdar S , Thompson W , Ahmad N , Gordon C , Addison C . The use and effectiveness of complementary and alternative medicine for pain in sickle cell anemia. Complement Ther Clin Pract. 2013;19:184‐187. 24199970 10.1016/j.ctcp.2013.05.003

[13] Armstrong AR , Thiebaut SP , Brown LJ , Nepal B . Australian adults use complementary and alternative medicine in the treatment of chronic illness: a national study. Aust N Z J Public Health. 2011;35:384‐390. 21806735 10.1111/j.1753-6405.2011.00745.x

[14] Amonkar SJ , Dunbar AM . Do patients and general practitioners have different perceptions about the management of simple mechanical back pain? Int Musculoskelet Med. 2011;33:3‐7.

[15] Skelton AM , Murphy EA , Murphy RJ , O'Dowd TC . Patients' views of low back pain and its management in general practice. Br J Gen Pract. 1996;46:153‐156. 8731620 PMC1239572

[16] Ghildayal N , Johnson PJ , Evans RL , Kreitzer MJ . Complementary and alternative medicine use in the US adult low back pain population. Glob Adv Health Med. 2016;5:69‐78. 26937316 10.7453/gahmj.2015.104 PMC4756777

[17] Schers H , Braspenning J , Drijver R , Wensing M , Grol R . Low back pain in general practice: reported management and reasons for not adhering to the guidelines in The Netherlands. Br Gen Pract. 2000;50:640‐644. PMC1313775 11042916

[18] National Institute For Health And Care Excellence . Non‐specific low back pain and sciatica: management (draft). 2016 https://www.nice.org.uk/guidance/indevelopment/gid-cgwave0681?unlid=957653473201610269250. Accessed September 25, 2016.

[19] Runciman WB , Hunt TD , Hannaford NA , et al. CareTrack: assessing the appropriateness of health care delivery in Australia. Med J Aust. 2012;197:100‐105. 22794056 10.5694/mja12.10510

[20] Henderson JV , Harrison CM , Britt HC , Bayram CF , Miller GC . Prevalence, causes, severity, impact, and management of chronic pain in Australian general practice patients. Pain Med. 2013;14:1346‐1361. 23855874 10.1111/pme.12195

[21] Williams CM , Maher CG , Hancock MJ , et al. Low back pain and best practice care: a survey of general practice physicians. Arch Intern Med. 2010;170:271‐277. 20142573 10.1001/archinternmed.2009.507

[22] Briggs AM , Slater H , Bunzli S , et al. Consumers' experiences of back pain in rural Western Australia: access to information and services, and self‐management behaviours. BMC Health Serv Res. 2012;12:357. 23057669 10.1186/1472-6963-12-357 PMC3494578

[23] Young CE , Boyle FM , Brooker KS , Mutch AJ . Incorporating patient preferences in the management of multiple long‐term conditions: is this a role for clinical practice guidelines? JOC. 2015;5:10. 10.15256/joc.2015.5.53 PMC5636037 29090160

[24] Walker BF , Muller R , Grant WD . Low back pain in Australian adults: the economic burden. Asia Pac J Public Health. 2003;15:79‐87. 15038680 10.1177/101053950301500202

[25] Wluka A , Chou L , Briggs A , Cicuttini F . Understanding the needs of consumers with musculoskeletal conditions: Consumers' perceived needs of health information, health services and other non‐medical services: a systematic scoping review. Melbourne, Vic: MOVE Muscle, Bone & Joint Health; 2016.

[26] Armstrong R , Hall BJ , Doyle J , Waters E . ‘Scoping the scope' of a cochrane review. Journal of Public Health. 2011;33:147‐150. 21345890 10.1093/pubmed/fdr015

[27] Levac D , Colquhoun H , O'Brien KK . Scoping studies: advancing the methodology. Implement Sci. 2010;5:69‐69. 20854677 10.1186/1748-5908-5-69 PMC2954944

[28] Asadi‐Lari M , Tamburini M , Gray D . Patients' needs, satisfaction, and health related quality of life: towards a comprehensive model. Health Qual Life Outcomes. 2004;2:32‐32. 15225377 10.1186/1477-7525-2-32 PMC471563

[29] (CASP) CASP . CASP Checklists (URL used) Oxford. CASP 2014.

[30] Hoy D , Brooks P , Woolf A , et al. Assessing risk of bias in prevalence studies: modification of an existing tool and evidence of interrater agreement. J Clin Epidemiol. 2012;65:934‐939. 22742910 10.1016/j.jclinepi.2011.11.014

[31] Walsh D , Downe S . Meta‐synthesis method for qualitative research: a literature review. J Adv Nurs. 2005;50:204‐211. 15788085 10.1111/j.1365-2648.2005.03380.x

[32] Yardley L , Dennison L , Coker R , et al. Patients' views of receiving lessons in the Alexander technique and an exercise prescription for managing back pain in the ATEAM trial. Fam Pract. 2010;27:198‐204. 20032168 10.1093/fampra/cmp093

[33] Campbell C , Guy A . `Why can't they do anything for a simple back problem?': a qualitative examination of expectations for low back pain treatment and outcome. J Health Psychol. 2007;12:641‐652. 17584815 10.1177/1359105307078171

[34] Cook FM , Hassenkamp AM . Active rehabilitation for chronic low back pain: the patient's perspective. Physiotherapy. 2000;86:61‐68.

[35] Cooper K , Smith BH , Hancock E . Patient‐centredness in physiotherapy from the perspective of the chronic low back pain patient. Physiotherapy. 2008;94:244‐252.

[36] Cooper K , Smith BH , Hancock E . Patients' perceptions of self‐management of chronic low back pain: evidence for enhancing patient education and support. Physiotherapy. 2009;95:43‐50. 19627685 10.1016/j.physio.2008.08.005

[37] Dean SG , Smith JA , Payne S , Weinman J . Managing time: an interpretative phenomenological analysis of patients' and physiotherapists' perceptions of adherence to therapeutic exercise for low back pain. Disabil Rehabil. 2005;27:625‐636. 16019873 10.1080/0963820500030449

[38] Dima A , Lewith GT , Little P , Moss‐Morris R , Foster NE , Bishop FL . Identifying patients' beliefs about treatments for chronic low back pain in primary care: a focus group study. Br J Gen Pract. 2013;63:e490‐e498. 23834886 10.3399/bjgp13X669211 PMC3693806

[39] Keen S , Dowell AC , Hurst K , Moffett JA , Tovey P , Williams R . Individuals with low back pain: how do they view physical activity? Fam Pract. 1999;16:39‐45. 10321394 10.1093/fampra/16.1.39

[40] May S . Patients' attitudes and beliefs about back pain and its management after physiotherapy for low back pain. Physiother Res Int. 2007;12:126‐135. 17624898 10.1002/pri.367

[41] May SJ . Patient satisfaction with management of back pain. Part 1: what is satisfaction? Review of satisfaction with medical management. Physiotherapy. 2001;87:4‐5.

[42] Pincus T , Vogel S , Savage R , Newman S . Patients' satisfaction with osteopathic and GP management of low back pain in the same surgery. Complement Ther Med. 2000;8:180‐186. 11068348 10.1054/ctim.2000.0378

[43] Westmoreland JL , Williams NH , Wilkinson C , Wood F , Westmoreland A . Should your GP be an osteopath? Patients' views of an osteopathy clinic based in primary care. Complement Ther Med. 2007;15:121‐127. 17544863 10.1016/j.ctim.2005.11.006

[44] Clarissa H , Sherman KJ , Eaves ER , et al. New perspectives on patient expectations of treatment outcomes: results from qualitative interviews with patients seeking complementary and alternative medicine treatments for chronic low back pain. BMC Altern Med. 2014;14:276‐285. 210p. 10.1186/1472-6882-14-276 PMC4129105 25077732

[45] Eaves ER , Sherman KJ , Ritenbaugh C , et al. A qualitative study of changes in expectations over time among patients with chronic low back pain seeking four CAM therapies. BMC Complement Alt Med. 2015;15:1‐12. 10.1186/s12906-015-0531-9 PMC4322442 25652396

[46] Allegretti A , Borkan J , Reis S , Griffiths F . Paired interviews of shared experiences around chronic low back pain: classic mismatch between patients and their doctors. Fam Pract. 2010;27:676‐683. 20671000 10.1093/fampra/cmq063

[47] Astin JA . Why patients use alternative medicine: results of a national study. J Am Med Assoc. 1998;279:1548‐1553. 10.1001/jama.279.19.1548 9605899

[48] Carey TS , Evans AT , Hadler NM , et al. Acute severe low back pain: a population‐based study of prevalence and care‐seeking. Spine. 1996;21:339‐344. 8742211 10.1097/00007632-199602010-00018

[49] Carey TS , Garrett J , Jackman A , et al. The outcomes and costs of care for acute low back pain among patients seen by primary care practitioners, chiropractors, and orthopedic surgeons. N Engl J Med. 1995;333:913‐917. 7666878 10.1056/NEJM199510053331406

[50] Carey TS , Garrett JM , Jackman A , Hadler N . Recurrence and care seeking after acute back pain: results of a long‐term follow‐up study. North Carolina Back Pain Project. Med Care. 1999;37:157‐164. 10024120 10.1097/00005650-199902000-00006

[51] Lyons KJ , Salsbury SA , Hondras MA , Jones ME , Andresen AA , Goertz CM . Perspectives of older adults on co‐management of low back pain by doctors of chiropractic and family medicine physicians: a focus group study. BMC Altern Med. 2013;13:225. 10.1186/1472-6882-13-225 PMC3847523 24040970

[52] Nyiendo J , Haas M , Goldberg B , Sexton G . Pain, disability, and satisfaction outcomes and predictors of outcomes: a practice‐based study of chronic low back pain patients attending primary care and chiropractic physicians. J Manipulative Physiol Ther. 2001;24:433‐439. 11562650

[53] Nyiendo J , Haas M , Goldberg B , Sexton G . Patient characteristics and physicians' practice activities for patients with chronic low back pain: a practice‐based study of primary care and chiropractic physicians. J Manipulative Physiol Ther. 2001;24:92‐100. 11208221 10.1067/mmt.2001.112565

[54] Sharma R , Haas M , Stano M . Patient attitudes, insurance, and other determinants of self‐referral to medical and chiropractic physicians. Am J Public Health. 2003;93:2111‐2117. 14652343 10.2105/ajph.93.12.2111 PMC1448161

[55] Sherman KJ , Cherkin DC , Ichikawa L , et al. Treatment expectations and preferences as predictors of outcome of acupuncture for chronic back pain. Spine. 2010;35:1471‐1477. 20535051 10.1097/BRS.0b013e3181c2a8d3 PMC2895682

[56] Chenot JF , Becker A , Leonhardt C , et al. Use of complementary alternative medicine for low back pain consulting in general practice: a cohort study. BMC Altern Med. 2007;7:42. 10.1186/1472-6882-7-42 PMC2222227 18088435

[57] Chenot JF , Leonhardt C , Keller S , et al. The impact of specialist care for low back pain on health service utilization in primary care patients: a prospective cohort study. Eur J Pain. 2008;12:275‐283. 17681811 10.1016/j.ejpain.2007.06.004

[58] Heyduck K , Meffert C , Glattacker M . Illness and treatment perceptions of patients with chronic low back pain: characteristics and relation to individual, disease and interaction variables. J Clin Psychol Med Settings. 2014;21:267‐281. 25100026 10.1007/s10880-014-9405-4

[59] Sigrell H . Expectations of chiropractic patients: the construction of a questionnaire. J Manipulative Physiol Ther. 2001;24:440‐444. 11562651

[60] Sigrell H . Expectations of chiropractic treatment: what are the expectations of new patients consulting a chiropractor, and do chiropractors and patients have similar expectations? J Manipulative Physiol Ther. 2002;25:300‐305. 12072850 10.1067/mmt.2002.124422

[61] Liddle SD , Gracey JH , Baxter GD . Advice for the management of low back pain: a systematic review of randomised controlled trials. Manual Ther. 2007;12:310‐327. 10.1016/j.math.2006.12.009 17395522

[62] Scheermesser M , Bachmann S , Schamann A , Oesch P , Kool J . A qualitative study on the role of cultural background in patients' perspectives on rehabilitation. BMC Musculoskelet Disord. 2012;13:1‐13. 22269636 10.1186/1471-2474-13-5 PMC3398320

[63] Medina‐Mirapeix F , Escolar‐Reina P , Gascón‐Cánovas JJ , Montilla‐Herrador J , Collins SM . Personal characteristics influencing patients' adherence to home exercise during chronic pain: a qualitative study. J Rehab Med. 2009;41:347‐352. 10.2340/16501977-0338 19363568

[64] Schers H , Wensing M , Huijsmans Z , van Tulder M , Grol R . Implementation barriers for general practice guidelines on low back pain a qualitative study. Spine. 2001;26:E348‐E353. 11474367 10.1097/00007632-200108010-00013

[65] Ferreira ML , Ferreira PH , Herbert RD , Latimer J . People with low back pain typically need to feel ‘much better' to consider intervention worthwhile: an observational study. Aust J Physiother. 2009;55:123‐127. 19463083 10.1016/s0004-9514(09)70042-x

[66] Grimmer K , Sheppard L , Pitt M , Magarey M , Trott P . Differences in stakeholder expectations in the outcome of physiotherapy management of acute low back pain. Int J Qual Health Care. 1999;11:155‐162. 10442846 10.1093/intqhc/11.2.155

[67] Slade SC , Molloy E , Keating JL . People with non‐specific chronic low back pain who have participated in exercise programs have preferences about exercise: a qualitative study. Aust J Physiother. 2009;55:115‐121. 19463082 10.1016/s0004-9514(09)70041-8

[68] Slade SC , Molloy E , Keating JL . `Listen to me, tell me': a qualitative study of partnership in care for people with non‐specific chronic low back pain. Clin Rehabil. 2009;23:270‐280. 19218301 10.1177/0269215508100468

[69] Slade SC , Molloy E , Keating JL . Stigma experienced by people with nonspecific chronic low back pain: a qualitative study. Pain Med. 2009;10:143‐154. 19222775 10.1111/j.1526-4637.2008.00540.x

[70] Crowe M , Whitehead L , Jo Gagan M , Baxter D , Panckhurst A . Self‐management and chronic low back pain: a qualitative study. J Adv Nurs. 2010;66:1478‐1486. 20492018 10.1111/j.1365-2648.2010.05316.x

[71] Borkan J , Reis S , Hermoni D , Biderman A . Talking about the pain: a patient‐centered study of low back pain in primary care. Soc Sci Med. 1995;40:977‐988. 7792636 10.1016/0277-9536(94)00156-n

[72] Chen LC , Cheng LJ , Zhang Y , He X , Knaggs RD . Acupuncture or low frequency infrared treatment for low back pain in chinese patients: a discrete choice experiment. PLoS One. 2015;5:e0126912. 10.1371/journal.pone.0126912 PMC4447362 26020251

[73] Baumann M , Euller‐Ziegler L , Guillemin F . Evaluation of the expectations osteoarthritis patients have concerning healthcare, and their implications for practitioners. Clin Exp Rheumatol. 2007;25:404‐409. 17631736

[74] Choi BKL , Verbeek JH , Tam WW‐S , Jiang JY . Exercises for prevention of recurrences of low‐back pain. Cochrane Database of Syst Rev. 2010;67:795‐796. 10.1002/14651858.CD006555.pub2 PMC8078403 20091596

[75] Rabey M , Beales D , Slater H , O'Sullivan P . Multidimensional pain profiles in four cases of chronic non‐specific axial low back pain: an examination of the limitations of contemporary classification systems. Man Ther. 2015;20:138‐147. 25153893 10.1016/j.math.2014.07.015

[76] Speerin R , Slater H , Li L , et al. Moving from evidence to practice: models of care for the prevention and management of musculoskeletal conditions. Best Pract Res Clin Rheumatol. 2014;28:479‐515. 25481427 10.1016/j.berh.2014.07.001

[77] Vibe Fersum K , O'Sullivan P , Skouen JS , Smith A , Kvale A . Efficacy of classification‐based cognitive functional therapy in patients with non‐specific chronic low back pain: a randomized controlled trial. Eur J Pain. 2013;17:916‐928. 23208945 10.1002/j.1532-2149.2012.00252.x PMC3796866

[78] Hill JC , Whitehurst DG , Lewis M , et al. Comparison of stratified primary care management for low back pain with current best practice (STarT Back): a randomised controlled trial. Lancet. 2011;378:1560‐1571. 21963002 10.1016/S0140-6736(11)60937-9 PMC3208163

[79] Bishop FL , Dima AL , Ngui J , et al. Lovely pie in the sky plans: a qualitative study of clinicians' perspectives on guidelines for managing low back pain in primary care in England. Spine (Phila Pa 1976). 2015; 40:1842‐1850. 26571064 10.1097/BRS.0000000000001215

[80] Synnott A , O'Keeffe M , Bunzli S , Dankaerts W , O'Sullivan P , O'Sullivan K . Physiotherapists may stigmatise or feel unprepared to treat people with low back pain and psychosocial factors that influence recovery: a systematic review. J Physiother. 2015;61:68‐76. 25812929 10.1016/j.jphys.2015.02.016

[81] Slade SC , Patel S , Psychol C , Underwood M , Keating JL . What are patient beliefs and perceptions about exercise for nonspecific chronic low back pain?: a systematic review of qualitative studies. Clin J Pain. 2014;30:995‐1005. 24300225 10.1097/AJP.0000000000000044

[82] LeFebvre R , Peterson D , Haas M . Evidence‐based practice and chiropractic care. J Evid‐Based Complementary Alternat Med. 2012;18:75‐79. 10.1177/2156587212458435 PMC3716373 23875117

[83] Rubinstein SM , Terwee CB , Assendelft WJ , de Boer MR , van Tulder MW . Spinal manipulative therapy for acute low‐back pain. Cochrane Database Syst Rev. 2012;9:CD008880. 10.1002/14651858.CD008880.pub2 PMC6885055 22972127

[84] Arthritis Research . Practitioner‐based complementary and alternative therapies for the treatment of rheumatoid arthritis, osteoarthritis, fibromyalgia and low back pain. United Kingdom 2013 http://www.arthritisresearchuk.org/arthritis-information/complementary-and-alternative-medicines/complementary-and-alternative-therapies.aspx.

[85] Millar WJ . Patterns of use–alternative health care practitioners. Health Rep. 2001;13:9‐21. 15069805

[86] Chaparro LE , Furlan AD , Deshpande A , Mailis‐Gagnon A , Atlas S , Turk DC . Opioids compared to placebo or other treatments for chronic low‐back pain. Cochrane Database Syst Rev. 2013;8:CD004959. 10.1002/14651858.CD004959.pub4 PMC11056234 23983011

[87] Engers AJ , Jellema P , Wensing M , van der Windt DAWM , Grol R , van Tulder MW . Individual patient education for low back pain. Cochrane Database Syst Rev. 2008;1:CD004057. 10.1002/14651858.CD004057.pub3 PMC6999124 18254037

[88] Enthoven WTM , Roelofs PDDM , Deyo RA , van Tulder MW , Koes BW . Non‐steroidal anti‐inflammatory drugs for chronic low back pain. Cochrane Database Syst Rev. 2016;2:CD012087. 26863524 10.1002/14651858.CD012087 PMC7104791

[89] Hayden J , van Tulder MW , Malmivaara A , Koes BW . Exercise therapy for treatment of non‐specific low back pain. Cochrane Database Syst Rev. 2005;3:CD000335. 10.1002/14651858.CD000335.pub2 PMC10068907 16034851

[90] Roelofs PDDM , Deyo RA , Koes BW , Scholten RJPM , van Tulder MW . Non‐steroidal anti‐inflammatory drugs for low back pain. Cochrane Database Syst Rev. 2008;1:CD000396. 10.1002/14651858.CD000396.pub3 PMC10220428 18253976

[91] Staal JB , de Bie R , de Vet HCW , Hildebrandt J , Nelemans P . Injection therapy for subacute and chronic low‐back pain. Cochrane Database Syst Rev. 2008;3:CD001824. 10.1002/14651858.CD001824.pub3 PMC7096223 18646078

[92] van Tulder MW , Touray T , Furlan AD , Solway S , Bouter LM . Muscle relaxants for non‐specific low‐back pain. Cochrane Database Syst Rev. 2003;2:CD004252. 10.1002/14651858.CD004252 PMC6464310 12804507

[93] Carr EC , Worswick L , Wilcock PM , Campion‐Smith C , Hettinga D . Improving services for back pain: putting the patient at the centre of interprofessional education. Qual Prim Care. 2012;20:345‐353. 23114002

[94] Andersson S , Sundberg T , Falkenberg E , He T . Patients' experiences and perceptions of integrative care for back and neck pain. Altern Ther Health Med. 2012;18:25‐32. 22875559

[95] Deyo RA . Real help and red herrings in spinal imaging. N Engl J Med. 2013;368:1056‐1058. 23484834 10.1056/NEJMe1215599

[96] Deyo RA , Weinstein JN . Low Back Pain. N Engl J Med. 2001;344:363‐370. 11172169 10.1056/NEJM200102013440508

[97] Ong BN , Konstantinou K , Corbett M , Hay E . Patients' own accounts of sciatica: a qualitative study. Spine (Phila Pa 1976). 2011;36:1251‐1256. 21343854 10.1097/BRS.0b013e318204f7a2

[98] Vroman K , Warner R , Chamberlain K . Now let me tell you in my own words: narratives of acute and chronic low back pain. Disabil Rehabil. 2009;31:976‐987. 19037775 10.1080/09638280802378017

[99] Walker J , Holloway I , Sofaer B . In the system: the lived experience of chronic back pain from the perspectives of those seeking help from pain clinics. Pain. 1999;80:621‐628. 10342423 10.1016/S0304-3959(98)00254-1

[100] Darlow B , Dowell A , Baxter GD , Mathieson F , Perry M , Dean S . The enduring impact of what clinicians say to people with low back pain. Ann Fam Med. 2013;11:527‐534. 24218376 10.1370/afm.1518 PMC3823723

[101] Toye F , Barker K . ‘Could I be imagining this?'‐The dialectic struggles of people with persistent unexplained back pain. Disabil Rehabil. 2010;32:1722‐1732. 20170277 10.3109/09638281003657857

[102] Hofstede SN , van Bodegom‐Vos L , Wentink MM , Vleggeert‐Lankamp CL , Vliet Vlieland TP , Marang‐van de Mheen PJ . Most important factors for the implementation of shared decision making in sciatica care: ranking among professionals and patients. PLoS One. 2014;9:e94176. 24710328 10.1371/journal.pone.0094176 PMC3978036

[103] Stenberg G , Fjellman‐Wiklund A , Ahlgren C . “Getting confirmation”: gender in expectations and experiences of healthcare for neck or back patients. J Rehabil Med. 2012;44:163‐171. 22234575 10.2340/16501977-0912

[104] McCarthy CJ , Oldham JA , Sephton R . Expectations and satisfaction of patients with low back pain attending a multidisciplinary rehabilitation service. Physiother Res Int. 2005;10:23‐31. 15991484 10.1002/pri.21

[105] Borenstein D . Mechanical low back pain[mdash]a rheumatologist's view. Nat Rev Rheumatol. 2013;9:643‐653. 24018549 10.1038/nrrheum.2013.133

[106] Ferreira ML , Machado G , Latimer J , Maher C , Ferreira PH , Smeets RJ . Factors defining care‐seeking in low back pain – a meta‐analysis of population based surveys. Eur J Pain. 2010;14:741.e1‐747.e7. 10.1016/j.ejpain.2009.11.005 20036168

[107] O'Sullivan PB , Beales DJ , Smith AJ , Straker LM . Low back pain in 17 year olds has substantial impact and represents an important public health disorder: a cross‐sectional study. BMC Pub Health. 2012;12:100. 22304903 10.1186/1471-2458-12-100 PMC3313872

[108] Beales D , Fried K , Nicholas M , Blyth F , Finniss D , Moseley GL . Management of musculoskeletal pain in a compensable environment: Implementation of helpful and unhelpful Models of Care in supporting recovery and return to work. Best Pract Res Clin Rheumatol. 2016;30:445‐467. 27886941 10.1016/j.berh.2016.08.011

[109] French SD , Bennell KL , Nicolson PJ , Hodges PW , Dobson FL , Hinman RS . What do people with knee or hip osteoarthritis need to know? An international consensus list of essential statements for osteoarthritis. Arthritis Care Res (Hoboken). 2015;67:809‐816. 25418120 10.1002/acr.22518

[110] Briggs AM , Jordan JE , O'Sullivan PB , et al. Individuals with chronic low back pain have greater difficulty in engaging in positive lifestyle behaviours than those without back pain: an assessment of health literacy. BMC Musculoskelet Disord. 2011;12:161. 21756363 10.1186/1471-2474-12-161 PMC3155909

[111] Walsh L , Hill S , Wluka A , et al. Harnessing and supporting consumer involvement in the development and implementation of Models of Care for musculoskeletal health. Best Pract Res Clin Rheumatol. 2016;30:420‐444. 27886940 10.1016/j.berh.2016.09.004

[112] Hinman RS , Nicolson PJ , Dobson FL , Bennell KL . Use of nondrug, nonoperative interventions by community‐dwelling people with hip and knee osteoarthritis. Arthritis Care Res (Hoboken). 2015;67:305‐309. 25048646 10.1002/acr.22395

