# Supplementary material for: Patients' perceived needs for allied health, and complementary and alternative medicines for low back pain: A systematic scoping review
Source: Health Expect. 2018 Jul 7;21(5):824–47. doi: 10.1111/hex.12676 (PMC6186543; doi:10.1111/hex.12676)
Supplement: Supplementary file 1 [file HEX-21-824-s001.docx]

| **AUTHOR, Year** | | | **CASP 1^1^** | | **CASP 2^2^** | | | **CASP 3^3^** | **CASP 4^4^** | | **CASP 5^5^** | **CASP 6^6^** | **CASP 7^7^** | **CASP 8^8^** | **CASP 9^9^** | **CASP10^10^** |
| --- | --- | --- | --- | --- | --- | --- | --- | --- | --- | --- | --- | --- | --- | --- | --- | --- |
| Allegretti, 2010([38](#_ENREF_38)) | | |  | |  | | |  |  | |  |  |  |  |  |  |
| Borkan, 1995([63](#_ENREF_63)) | | |  | |  | | |  |  | |  |  |  |  |  |  |
| Campbell, 2007([24](#_ENREF_24)) | | |  | |  | | |  |  | |  |  |  |  |  |  |
| Cook, 2000([25](#_ENREF_25)) | | |  | |  | | |  |  | |  |  |  |  |  |  |
| Cooper, 2008([26](#_ENREF_26)) | | |  | |  | | |  |  | |  |  |  |  |  |  |
| Cooper, 2009([27](#_ENREF_27)) | | |  | |  | | |  |  | |  |  |  |  |  |  |
| Crowe, 2010([62](#_ENREF_62)) | | |  | |  | | |  |  | |  |  |  |  |  |  |
| Dean, 2005([28](#_ENREF_28)) | | |  | |  | | |  |  | |  |  |  |  |  |  |
| Dima, 2013([29](#_ENREF_29)) | | |  | |  | | |  |  | |  |  |  |  |  |  |
| Eaves, 2015 ([37](#_ENREF_37)) | | |  | |  | | |  |  | |  |  |  |  |  |  |
| Grimmer, 1999([58](#_ENREF_58)) | | |  | |  | | |  |  | |  |  |  |  |  |  |
| Hsu, 2014([36](#_ENREF_36)) | | |  | |  | | |  |  | |  |  |  |  |  |  |
| Keen, 1999([30](#_ENREF_30)) | | |  | |  | | |  |  | |  |  |  |  |  |  |
| Liddle, 2007([53](#_ENREF_53)) | | |  | |  | | |  |  | |  |  |  |  |  |  |
| Lyons, 2013([43](#_ENREF_43)) | | |  | |  | | |  |  | |  |  |  |  |  |  |
| May, 2001([32](#_ENREF_32)) | | |  | |  | | |  |  | |  |  |  |  |  |  |
| May, 2007([31](#_ENREF_31)) | | |  | |  | | |  |  | |  |  |  |  |  |  |
| Medina-Mirapeix, 2009([55](#_ENREF_55)) | | |  | |  | | |  |  | |  |  |  |  |  |  |
| Scheermesser, 2012([54](#_ENREF_54)) | | |  | |  | | |  |  | |  |  |  |  |  |  |
| Schers, 2001([56](#_ENREF_56)) | | |  | |  | | |  |  | |  |  |  |  |  |  |
| Skelton, 1996 ([34](#_ENREF_34)) | | |  | |  | | |  |  | |  |  |  |  |  |  |
| Slade, 2009 ([60](#_ENREF_60)) | | |  | |  | | |  |  | |  |  |  |  |  |  |
| Slade, 2009 ([59](#_ENREF_59)) | | |  | |  | | |  |  | |  |  |  |  |  |  |
| Slade, 2009 ([61](#_ENREF_61)) | | |  | |  | | |  |  | |  |  |  |  |  |  |
| Westmoreland, 2007([35](#_ENREF_35)) | | |  | |  | | |  |  | |  |  |  |  |  |  |
| Yardley, 2010([22](#_ENREF_22)) | | |  | |  | | |  |  | |  |  |  |  |  |  |
| Legend: |  | Yes |  | No | |  | Can’t tell | | |  |  |  |  |  |  |  |

Figure 1. CASP tool for qualitative studies

^1^CASP 1: Was there a clear statement of the aims of the research

^2^CASP 2: Is a qualitative methodology appropriate?

^3^CASP 3:Was the research design appropriate to address the aims of the research?

^4^CASP 4: Was the recruitment strategy appropriate to the aims of the research?

^5^CASP 5: Was the data collected in a way that addressed the research issue?

^6^CASP 6: Has the relationship between researcher and participants been adequately considered?

^7^CASP 7: Have ethical issues been taken into consideration?

^8^CASP 8: Was the data analysis sufficiently rigorous?

^9^CASP 9: Is there a clear statement of findings?

^10^CASP 10: How valuable is the research?

| **Author, Year** | | | **Criteria 1^1^** | | | **Criteria 2^2^** | **Criteria 3^3^** | **Criteria 4^4^** | **Criteria 5^5^** | **Criteria 6^6^** | **Criteria 7^7^** | **Criteria 8^8^** | **Criteria 9^9^** | **Criteria10^10^** | **Overall risk of bias** |
| --- | --- | --- | --- | --- | --- | --- | --- | --- | --- | --- | --- | --- | --- | --- | --- |
| Amonkar, 2011([23](#_ENREF_23)) | | |  | | |  |  |  |  |  |  |  |  |  | High |
| Astin, 1998([39](#_ENREF_39)) | | |  | | |  |  |  |  |  |  |  |  |  | Moderate |
| Carey, 1995([41](#_ENREF_41)) | | |  | | |  |  |  |  |  |  |  |  |  | Moderate |
| Carey, 1996([40](#_ENREF_40)) | | |  | | |  |  |  |  |  |  |  |  |  | Moderate |
| Carey, 1999([42](#_ENREF_42)) | | |  | | |  |  |  |  |  |  |  |  |  | Moderate |
| Chen, 2015([64](#_ENREF_64)) | | |  | | |  |  |  |  |  |  |  |  |  | High |
| Chenot, 2007([48](#_ENREF_48)) | | |  | | |  |  |  |  |  |  |  |  |  | High |
| Chenot, 2008([49](#_ENREF_49)) | | |  | | |  |  |  |  |  |  |  |  |  | High |
| Ferreira, 2009([57](#_ENREF_57)) | | |  | | |  |  |  |  |  |  |  |  |  | High |
| Heyduck, 2014([50](#_ENREF_50)) | | |  | | |  |  |  |  |  |  |  |  |  | High |
| Nyiendo, 2000([45](#_ENREF_45)) | | |  | | |  |  |  |  |  |  |  |  | Moderate | Moderate |
| Nyiendo, 2001([44](#_ENREF_44)) | | |  | | |  |  |  |  |  |  |  |  |  | High |
| Pincus, 2000([33](#_ENREF_33)) | | |  | | |  |  |  |  |  |  |  |  |  | High |
| Sharma, 2003([46](#_ENREF_46)) | | |  | | |  |  |  |  |  |  |  |  |  | High |
| Sherman, 2004([10](#_ENREF_10)) | | |  | | |  |  |  |  |  |  |  |  |  | High |
| Sherman, 2010([47](#_ENREF_47)) | | |  | | |  |  |  |  |  |  |  |  |  | High |
| Sigrell, 2002([52](#_ENREF_52)) | | |  | | |  |  |  |  |  |  |  |  |  | High |
| Sigrell, 2004 ([51](#_ENREF_51)) | | |  | | |  |  |  |  |  |  |  |  |  | High |
| Yardley, 2010([22](#_ENREF_22)) | | |  | | |  |  |  |  |  |  |  |  |  | High |
| Legend: |  | Yes | |  | No | | | | | | | | | |  |

Figure 2. Hoy et al’s Risk of Bias tool for quantitative studies

^1^Criteria 1:Was the study’s target population a close representation of the national population in relation to relevant variables?

^2^Criteria 2: Was the sampling frame a true or close representation of the target population?

^3^Criteria 3: Was some form of random selection used to select the sample OR was a census taken?

^4^Criteria 4: Was the likelihood of nonresponse bias minimal?

^5^Criteria 5: Were data collected directly from the subjects?

^6^Criteria 6: Was an acceptable case definition used in the study?

^7^Criteria 7: Was the study instrument that measured the parameter of interest shown to have validity and reliability?

^8^Criteria 8: Was the same mode of data collection used for all subjects?

^9^Criteria 9: Was the length of the shortest prevalence period for the parameter of interest appropriate?

^10^Criteria 10: Were the numerator(s) and denominator(s) for the parameter of interest appropriate?

**Supplementary Appendix – Search Strategy**

Database: Ovid MEDLINE(R) 1946 to Present with Daily Update

Search Strategy:

--------------------------------------------------------------------------------

1 exp Back Pain/

2 exp Low Back Pain/

3 low back pain.tw.

4 backache.tw.

5 back pain.tw.

6 backpain.tw.

7 coccyx.tw.

8 coccydynia.tw.

9 dorsalgia.tw.

10 (lumbar adj3 pain).tw.

11 lumbago.tw.

12 sciatica.tw.

13 sciatic neuropathy/

14 sciatica/

15 spondylosis.tw.

16 exp Spondylosis/

17 1 or 2 or 3 or 4 or 5 or 6 or 7 or 8 or 9 or 10 or 11 or 12 or 13 or 14 or 15 or 16

18 1 or 2 or 3 or 4 or 5 or 6 or 7 or 8 or 9 or 10 or 11 or 12 or 13 or 14 or 15 or 16

***************************

Database: Ovid MEDLINE(R) 1946 to Present with Daily Update

Search Strategy:

--------------------------------------------------------------------------------

1 (consumer* or patient* or client* or customer* or service user*).tw.

2 patients/ or inpatients/ or outpatients/

3 1 or 2

4 (rheumatolog* or doctor* or physician* or practitioner* or clinician* or specialist* or consultant* or health professional* or nurs* or allied health or physiotherap* or physical therap* or chiropract* or occupational therap* or podiatr* or nutrition* or diet* or rehabilitat* or pain management).tw.

5 health personnel/ or allied health personnel/ or nutritionists/ or physical therapist assistants/ or physical therapists/ or exp medical staff/ or exp nurses/ or exp physicians/

6 Rheumatology/

7 Manipulation, Chiropractic/ or Chiropractic/

8 nutrition therapy/ or diet therapy/ or caloric restriction/ or diet, carbohydrate-restricted/ or diet, fat-restricted/ or diet, reducing/

9 Counseling/

10 Psychology/

11 Dietetics/

12 Podiatry/

13 Rehabilitation Nursing/

14 Nursing Care/

15 Rehabilitation/

16 Pain Management/

17 ((conservative or surgical or orthop?edic or complementary or traditional or ayurvedic or acupuncture or chinese or herbal or moxibustion or homeopath*) adj3 (medicine* or therap* or treatment* or management)).tw.

18 complementary therapies/ or acupuncture therapy/ or acupuncture analgesia/ or moxibustion/ or homeopathy/ or medicine, traditional/ or medicine, chinese traditional/

19 ((exercis* or hyperthermia induc* or short wave or ultra* or ambulatory or rehab* or self help or electr* or manipulat* or manual* or heat) adj5 (therap* or modalit* or treatment*)).tw.

20 physical therapy modalities/ or electric stimulation therapy/ or exercise therapy/ or hyperthermia, induced/ or short-wave therapy/ or ultrasonic therapy/

21 "Physical and Rehabilitation Medicine"/

22 (tens or transcutaneous electric nerve stimulation).tw.

23 transcutaneous electric nerve stimulation/

24 (stretch* or strength* or mobili*).tw.

25 muscle stretching exercises/ or resistance training/

26 Manipulation, Orthopedic/

27 Musculoskeletal Manipulations/

28 ((joint* or knee* or hip*) adj3 (replac* or prosthe*)).tw.

29 (arthroplast* or hemiarthroplast*).tw.

30 arthroplasty/ or arthroplasty, replacement/ or arthroplasty, replacement, hip/ or arthroplasty, replacement, knee/ or hemiarthroplasty/ or arthroscopy/

31 ((anti-inflammatory or antiinflammatory or analgesic) adj3 (agent* or drug* or medic*)).tw.

32 ((nonsteroid* anti-inflammatory or nonsteroid* antiinflammatory or non steroid* anti-inflammatory or non steroid* antiinflammatory) adj (agent* or drug* or medic*)).tw.

33 pain killer*.tw.

34 analgesics/ or analgesics, non-narcotic/ or acetaminophen/ or ibuprofen/ or exp anti-inflammatory agents, non-steroidal/ or analgesics, short-acting/

35 Analgesics, Opioid/

36 steroid*.tw.

37 Steroids/

38 Prednisolone/

39 (disease modifying anti rheumatic adj (agent* or drug* or medic*)).tw.

40 antirheumatic agents/ or azathioprine/ or chloroquine/ or gold sodium thiomalate/ or gold sodium thiosulfate/ or hydroxychloroquine/ or methotrexate/ or sulfasalazine/

41 Biological Products/

42 Tumor Necrosis Factors/

43 Tumor Necrosis Factor-alpha/

44 Interleukin 1 Receptor Antagonist Protein/

45 Infliximab.tw.

46 Etanercept.tw.

47 Certolizumab.tw.

48 Golimumab.tw.

49 Interleukin 1 inhibitor.tw.

50 Anakinra.tw.

51 Canakinumab.tw.

52 Interleukin 6.tw.

53 Tocilizumab.tw.

54 CD-20.tw.

55 Rituximab.tw.

56 Co-stimulatory blockade.tw.

57 Abatacept.tw.

58 biologic*.tw.

59 tnf.tw.

60 Diphosphonates/

61 Bisphosphonate*.tw.

62 Vitamin D/

63 Cholecalciferol/

64 vitamin D.tw.

65 Calcium/

66 Calcium.tw.

67 self-help devices/ or wheelchairs/

68 exp Dependent Ambulation/

69 canes/ or crutches/ or orthotic devices/ or braces/ or walkers/

70 (walking adj3 (cane* or frame* or aid*)).tw.

71 self help devices.tw.

72 assistive devices.tw.

73 or/4-72

74 (utili* or need* or seek* or retriev* or provid* or provision or source* or aid* or promot* or access* or demand* or insufficien* or deficit* or gap* or barrier* or enabler* or facilitat* or deliver* or implement* or manag* or coordinat*).tw.

75 Needs Assessment/ or "Health Services Needs and Demand"/ or Health Services Accessibility/

76 74 or 75

77 ((consumer* or patient* or client* or customer* or service user*) adj4 (need* or want* or like* or interest* or prefer* or satisf* or perspective* or experience* or attitude* or belief* or practice* or concern* or support* or participat* or advoca* or center* or centr* or orient* or focus* or empower* or expect* or opinion* or view* or perceive* or perception* or tailor* or bespoke or involv* or priorit* or control*)).tw.

78 "patient acceptance of health care"/ or patient preference/ or patient satisfaction/ or Patient-Centered Care/ or Health Knowledge, Attitudes, Practice/

79 77 or 78

80 ((household or out of pocket) adj3 expen*).tw.

81 "cost of illness"/ or health expenditures/ or exp "fees and charges"/

82 Waiting Lists/

83 Rural Health/ or Rural Population/

84 Urban Health/ or Urban Population/

85 Primary Health Care/

86 secondary care/ or tertiary healthcare/

87 Vulnerable Populations/

88 exp Culture/

89 communication barriers/

90 (cost* or fee* or charge* or expen* or wait* or time* or rural* or remote* or urban* or primary or secondary or tertiary or acute* or cultur* or communicat* or language* or linguistic*).tw.

91 80 or 81 or 82 or 83 or 84 or 85 or 86 or 87 or 88 or 89 or 90

92 3 and 73 and 76 and 79 and 91

93 78 and 92

***************************
